# Redefining the architecture of ferlin proteins: Insights into multi-domain protein structure and function

**DOI:** 10.1371/journal.pone.0270188

**Published:** 2022-07-28

**Authors:** Matthew J. Dominguez, Jon J. McCord, R. Bryan Sutton

**Affiliations:** 1 Department of Cell Physiology and Molecular Biophysics, Texas Tech University Health Sciences Center, Lubbock, TX, United States of America; 2 Center for Membrane Protein Research, Texas Tech University Health Sciences Center, Lubbock, TX, United States of America; Consejo Superior de Investigaciones Cientificas, SPAIN

## Abstract

Ferlins are complex, multi-domain proteins, involved in membrane trafficking, membrane repair, and exocytosis. The large size of ferlin proteins and the lack of consensus regarding domain boundaries have slowed progress in understanding molecular-level details of ferlin protein structure and function. However, *in silico* protein folding techniques have significantly enhanced our understanding of the complex ferlin family domain structure. We used RoseTTAFold to assemble full-length models for the six human ferlin proteins (dysferlin, myoferlin, otoferlin, Fer1L4, Fer1L5, and Fer1L6). Our full-length ferlin models were used to obtain objective domain boundaries, and these boundaries were supported by AlphaFold2 predictions. Despite the differences in amino acid sequence between the ferlin proteins, the domain ranges and distinct subdomains in the ferlin domains are remarkably consistent. Further, the RoseTTAFold/AlphaFold2 *in silico* boundary predictions allowed us to describe and characterize a previously unknown C2 domain, ubiquitous in all human ferlins, which we refer to as C2-FerA. At present, the ferlin domain-domain interactions implied by the full-length *in silico* models are predicted to have a low accuracy; however, the use of RoseTTAFold and AlphaFold2 as a domain finder has proven to be a powerful research tool for understanding ferlin structure.

## Introduction

Ferlins are large, multi-domain proteins that mediate Ca^2+^-dependent phospholipid interactions in multiple cell types [1]. Six paralogous ferlin genes have been annotated within the human genome: dysferlin (Fer1L1), otoferlin (Fer1L2), myoferlin (Fer1L3), Fer1L4, Fer1L5, and Fer1L6 [1]. Three of the ferlin proteins, dysferlin, otoferlin, and myoferlin, have well-studied biological functions. Dysferlin is an important component in skeletal muscle membrane repair, and mutations have been implicated in various muscular dystrophies [2–4]. Otoferlin is expressed in the brain and in vestibular hair cells in the ear [5], and is involved in Ca^2+^-dependent exocytosis in hair cells [6, 7]. Mutations in the otoferlin gene have been linked to sensorineural hearing loss (auditory neuropathy autosomal recessive 1) and non-syndromic sensorineural hearing (Autosomal recessive deafness 9) loss in humans [8]. Myoferlin is required for normal muscle cell development [9] and has established links to metastatic cancers [10, 11]. Fer1L4 has been categorized as a pseudogene in humans, and it may only function as a long noncoding RNA (lncRNA) [12–17]; however, Fer1L4 may act as a functional protein in mice and zebrafish [1]. There is some evidence that Fer1L5 [18–20] and Fer1L6 [21–23] are active proteins in humans, but the exact function of these ferlins is currently unknown.

The defining feature of ferlin proteins is their multiple tandem C2 domains. The prototypical C2 domain is approximately 130 residues in length and possesses eight *β*-strands that fold around a central Greek key motif with two *β*-sheets that form a *β*-sandwich [24]. There are at least two known folding topologies for C2 domains, Type-I (Type-S) and Type-II (Type-P) [25]. Three loops at the apex of the domain form a cup-like Ca^2+^ binding pocket and are labeled loops 1, 2, and 3 [26]. While not all C2 domains bind Ca^2+^, the C2 domains that do bind Ca^2+^, such as the two tandem C2 domains of synaptotagmin-1, possess up to six conserved acidic residues within the binding pocket with which to coordinate divalent cations [27]. Hydrophobic residues at the apices of loops 1 and 3 of the Ca^2+^ binding pocket form the basis of the Ca^2+^-dependent peripheral phospholipid interaction that is characteristic of many C2 domains [28].

**Fig 1:**
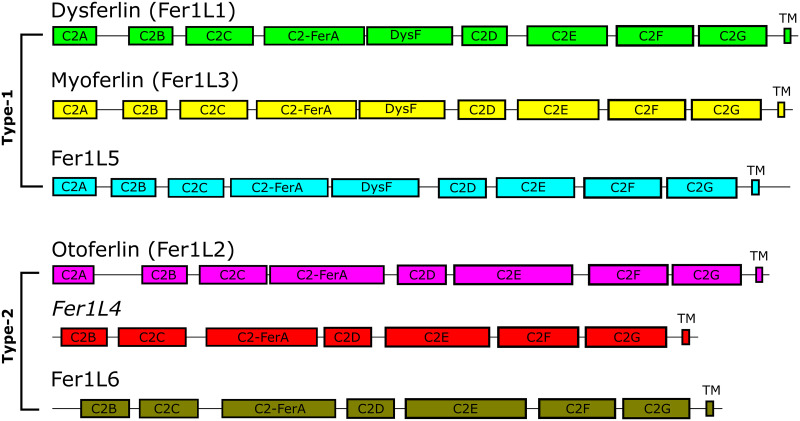
Scaled schematic diagram of the human ferlin family members. The length of the total protein is in proportion to the length of each domain. The six ferlin proteins are segregated by type. Type-1 ferlins possess a DysF domain; Type-2 ferlins lack a DysF domain [37]. ‘TM’ indicates the transmembrane span. *Fer1L4 in humans is currently characterized as a pseudogene.

**Fig 2:**
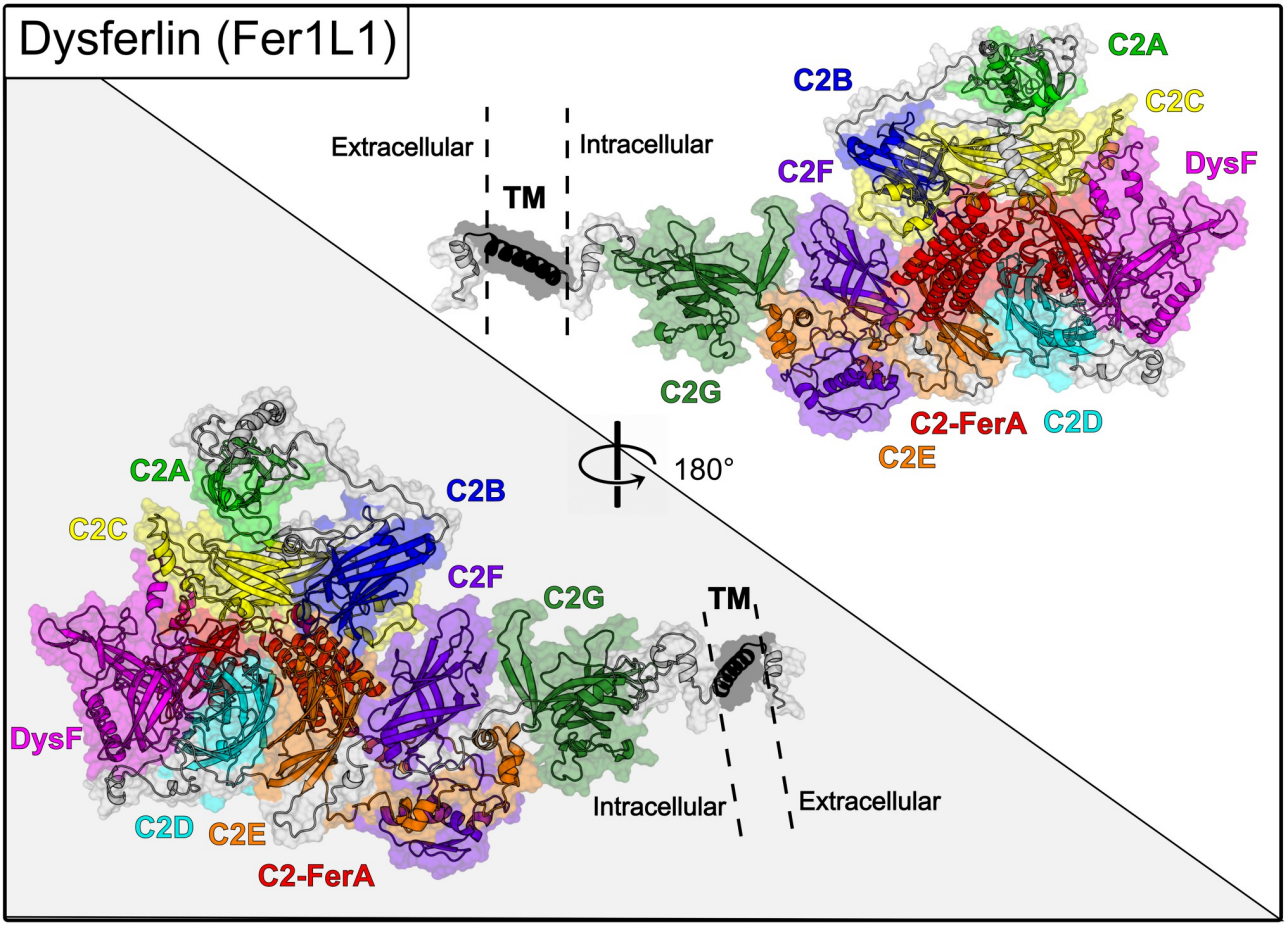
RoseTTAFold model of the full-length dysferlin. The full-length model indicated super-tertiary [53] domain interactions in the dysferlin model. The RoseTTAFold models that were used in this study were flexibly aligned using FATCAT [54]. Inconsistencies in the 3D models that were generated as a result of the elastic alignment process were repaired using PyMod [55]. Figures were rendered with PyMol and displayed as 180° views of the model. The various domains of dysferlin are shown as colored surfaces and similarly colored labels. The other ferlin full-length models can be found in the supplemental information (S1-S17 Figs and S1-S7 Tables in S1 File).

Early analysis of dysferlin’s primary sequence concluded that there were at least two C2 domains in the protein [29]. Later analyses extended the complexity of the ferlins to include six to seven C2 domains of mixed topology [30, 31]. After these initial estimates, several studies published differing domain boundaries and topologies for the C2 domains of dysferlin [32–34]. The assumptions used to establish the early dysferlin domain definitions were based on the known fold of the classical C2 domains such as synaptotagmin-1 C2A and C2B; however, primary and secondary sequence alignments used to determine domain boundaries could not precisely rationalize synaptotagmin-like C2 domain boundaries in all ferlin C2 domains. The synaptotagmin-like C2 domain assumption for ferlin proteins led to domain predictions with an incorrect number of *β*-strands per C2 domain and ferlin molecules dominated by unstructured linker sequences. With the advent of AlphaFold2 and RoseTTAFold [35, 36], we can now accurately compute unbiased C2 domain boundaries in large complex proteins such as ferlins (Fig 1). Furthermore, previously unknown domains can be defined within these proteins. While the domain-domain interactions for the full-length models of ferlin proteins using AlphaFold2 and RoseTTAFold are likely not yet accurate (Fig 2, S1-S17 Figs and S1-S7 Tables in S1 File), these two complementary methods make excellent domain finders that can produce experimentally testable domain boundaries to study complex multi-domain proteins such as ferlins.

## Materials and methods

### Model building and domain definitions

Overlapping primary sequences of each of the ferlin family members were submitted to the Robetta server for the structural assessment using the RoseTTAFold algorithm [36]. Assuming that there is little to no cooperative folding between domains, the amino acid sequence of each domain would be expected to fold independently [38, 39], thereby providing an internal control for the folding results. Overlapping domains were superimposed, and a model of the entire molecule was assembled using Modeller [40]. We utilized canonical protein sequences from the UniProt database (dysferlin, O75923; myoferlin, Q9NZM1; otoferlin, Q9HC10; Fer1L4, A9Z1Z3; Fer1L5, A0AVI2; and Fer1L6, Q2WGJ9). The final assembled protein models were relaxed using PyRosetta to optimize side-chain interactions (Fig 2, S1-S17 Figs and S1-S7 Tables in S1 File). The energy minimization script used is reproduced in the supplementary section (S1-S17 Figs and S1-S7 Tables in S1 File).

C2 domains are defined as protein domains possessing eight *β*-strands that fold with a characteristic C2 domain topology [41]. The boundary condition for each C2 domain in each modeled ferlin protein is defined as a range of residues that begin with the first residue that contributes to the backbone hydrogen bond pattern of the first *β*-strand of the domain through to the last residue that contributes a backbone hydrogen bond to the eighth *β*-strand of the domain. Loops that significantly deviate from the typical Type-II C2 pattern, as defined by the C2A domain of dysferlin, and are unique to the ferlin family were defined as subdomains. A more extensive definition of each subdomain is provided in the results section. A table with ferlin domain boundaries is in the supplementary section (S1-S17 Figs and S1-S7 Tables in S1 File).

### Molecular dynamics

Molecular dynamics (MD) systems were prepared using Charmm-GUI [42] to regularize the geometry of the extracted C2 domain models from the full-length ferlin molecules. Individual domains were simulated in an explicit water box using NAMD [43]. The MD was run for 10 ns to relieve clashes and to improve any nonideal stereochemistry that resulted from the model building process. The CHARMM36m force field was used throughout. Simulations of the DysF regions of dysferlin, myoferlin, and Fer1L5 were performed using WYF parameters for pi-cation interactions. The protein systems were solvated in a cubic water box (having basis vector lengths of ∼ 80 Å) under periodic boundary conditions using the TIP3 water model. The total charge of the system was neutralized by randomly substituting water molecules with K^+^ and Cl^-^, in addition to a 0.15 M overall salt concentration. The initial energy minimization was performed for 10,000 steps to avoid any bad contacts generated while solvating the system. A switching function was applied to the van der Waal’s potential energy from 10 to 12 Å to calculate nonbonded interactions. The Particle Mesh Ewald (PME) algorithm was used to calculate electrostatic interactions. Equilibration runs used the NVT ensemble at 300 K. Molecular dynamics production runs were performed under NPT conditions at 303.15 K. The domain models were extracted and analyzed using VMD.

### Model assessment

Ten aligned snapshots were extracted from the MD simulations. From the set of 10 snapshots, a representative model of each domain was selected based on its near-zero QMEAN Z-score as computed by SWISS-MODEL [44]. Violations in Ramachandran angles, clash scores, and the MolProbity scores for each predicted domain are reported in Supplementary Data (S1-S17 Figs and S1-S7 Tables in S1 File).

### Model-based primary sequence analysis

The structures and models of the known C2 domains of ferlins were superimposed and aligned using PROMALS3D [45]. The primary sequence alignment from PROMALS3D was used to compute the mean similarity and identity fractions using the SIAS server (http://imed.med.ucm.es/Tools/sias.html) for each domain (S1-S17 Figs and S1-S7 Tables in S1 File). Alignment graphics in the Supplementary Material were prepared using ESPript [46].

### Cloning, expression, purification, and characterization of C2-FerA

A codon-optimized human dysferlin cDNA gene was purchased from Addgene (Plasmid 67878) [47]. The DNA sequence corresponding to the human dysferlin C2-FerA domain (residues 588–868) was extracted from the dysferlin cDNA using the In-Fusion^®^ Snap Assembly kit and subcloned into a pET28a expression vector with an N-terminal 6 x His-tagged maltose-binding protein (MBP) fusion tag and a tobacco etch virus (TEV) cleavage site. The human dysferlin C2-FerA domain expression plasmid was transformed into Mix & Go BL21 Gold (DE3) competent cells prepared using Zymo Research’s Mix & Go! *E.coli* Transformation Kit and Buffer Set (T3002). The plasmid/competent cell mixture was plated on LB-agar plates supplemented with 50 *μ*g/mL kanamycin and incubated at 37 ℃ overnight. Multiple colonies were used to inoculate 100 mL of Luria Broth (LB) containing 50 *μ*g/mL kanamycin in a 250 mL baffled Fernbach flask. The inoculated media was grown in a shaking incubator at 250 rpm and 37 ℃ for 12–16 hours. 15 mL of the LB starter culture was used to inoculate a 2800 mL baffled Fernbach flask containing 1 L of terrific broth (TB) supplemented with 50 *μ*g/mL kanamycin. The inoculated media was grown in a shaking incubator at 250 rpm and 37 ℃ for four hours or until the culture reached an OD_600_ of at least 2.0. Next, the shaking incubator was cooled to 18 ℃, and protein expression was induced by the addition of 400 *μ*L of 1 M IPTG. After induction, the culture was grown for an additional 24 hours in the shaking incubator (250 rpm and 18 ℃). The culture with the expressed dysferlin C2-FerA domain was harvested by centrifugation and the cell pellets were flash-frozen in liquid nitrogen and stored at -80 ℃ until needed.

To begin the purification, cells were thawed in lysis buffer (40 mM HEPES pH 7.4, 150 mM NaCl), ruptured using a Microfluidics M-110EH microfluidizer, and cell debris was pelleted using a Beckman JA-20 rotor at 19,500 rpm and 4 ℃ for 1 hour. The clarified supernatant was applied to a 10 mL Ni-IDA (His6-Ni Superflow Resin) affinity column, which was equilibrated with lysis buffer, and incubated at 4 ℃ overnight. The supernatant was allowed to pass through the column and 200 mL lysis buffer including 20 mM imidazole was used to wash the column. Finally, human dysferlin C2-FerA was eluted with 35 mL lysis buffer including 250 mM imidazole. The resulting fusion protein was cleaved with 4 mg of Tobacco Etch Virus (TEV) protease at 4 ℃ overnight. Next, the TEV-cleaved protein was loaded onto a 30 mL GE-Healthcare Q-Sepharose Fast Flow^®^ column using a Bio-Rad NGC chromatography system. The TEV protein did not bind and most of the MBP did not bind to the Q-Sepharose^®^ resin. C2-FerA was eluted from the ion-exchange resin by applying a gradient from 0 M to 1 M NaCl over 30 minutes at a flow rate of 5 mL/min. The fractions containing the dysferlin C2-FerA domain were pooled and concentrated 1–0.5 mL using an Amicon^®^Ultra-15 Centrifugal Filter Unit with a 10 kDa molecular-weight cut off (UFC901024). The concentrated protein was further purified by size exclusion chromatography using Superdex-75 resin. Purity was assessed using SDS PAGE Any-kD Mini-PROTEAN TGX Stain-Free gels from Bio-Rad and protein concentration was quantified using absorbance at 280 nm and the calculated extinction coefficient of 46870 M^-1^ cm^-1^ from ProtParam on the ExPASy web server [48] (**Fig 7A**).

Purified dysferlin C2-FerA was buffer exchanged using a disposable PD 10 desalting column (Cytiva 17–0851-01) into a 10 mM sodium phosphate pH 7.4 buffer for circular dichroism (CD) spectroscopy. The CD spectrum was collected from 250–180 nm with 4 *μ*M C2-FerA using a J-850 spectropolarimeter from JASCO Corp. Data collection was performed at room temperature with an acquisition rate of 1 nm/sec and a data pitch of 0.1 nm. Additionally, CD spectra of 10 mM sodium phosphate pH 7.4 alone were collected and subtracted from the C2-FerA CD spectra to produce the final CD spectra used for fitting. The C2-FerA buffer-subtracted CD spectra were normalized to mean residue ellipticity and the secondary structure was estimated using the BeStSel webserver [49, 50]. We used the PDBMD2CD webserver [51] to calculate CD spectra based on the human dysferlin C2-FerA model to obtain an estimated secondary structure. The calculated values obtained from PDBMD2CD were then compared with the experimental CD spectra and secondary structure estimates (**Fig 7B**).

## Results

### Overall ferlin domain structure

Ferlin proteins have generally been thought of as flexible proteins that interact with Ca^2+^ and phospholipid membranes without a defined 3D structure [34]. In fact, most depictions of the full-length ferlin proteins display the structure as a series of boxes separated by long lines [34, 52]. The high proline and glycine content of the predicted linker regions between predicted domains suggested that conclusion. Surprisingly, RoseTTAFold and AlphaFold2 favored folding the ferlin molecules into elongated globular structures, typical for the super-tertiary structure of multi-domain proteins [53]. However, a super-tertiary structure has never been proposed for the ferlin protein family. The super-tertiary packing of the full-length ferlin protein models is roughly the shape of a prolate spheroid (American football). (Fig 2, S1-S17 Figs and S1-S7 Tables in S1 File).

An early prediction of the C2 domain topology in dysferlin suggested the presence of both Type-I and Type-II C2 domains [31]. The incorrect prediction of mixed C2 domain topologies likely had to do with the difficulty in fitting most of the domains into known C2 domain secondary structure patterns. The full-length ferlin models, using both RoseTTAFold and AlphaFold2, predict that all C2 domains of ferlin proteins are Type-II.

Interestingly, our ferlin models also show long conserved insertions within the predicted loops of many of the C2 domains. We refer to these loop insertions as subdomains. Here, we define subdomains as any C2 domain loop insertion greater than 20 amino acids (relative to the reference dysferlin C2A domain) that possess conserved secondary structural elements across at least two human ferlin paralogs.

### Analysis of ferlin C2A domains

**Fig 3:**
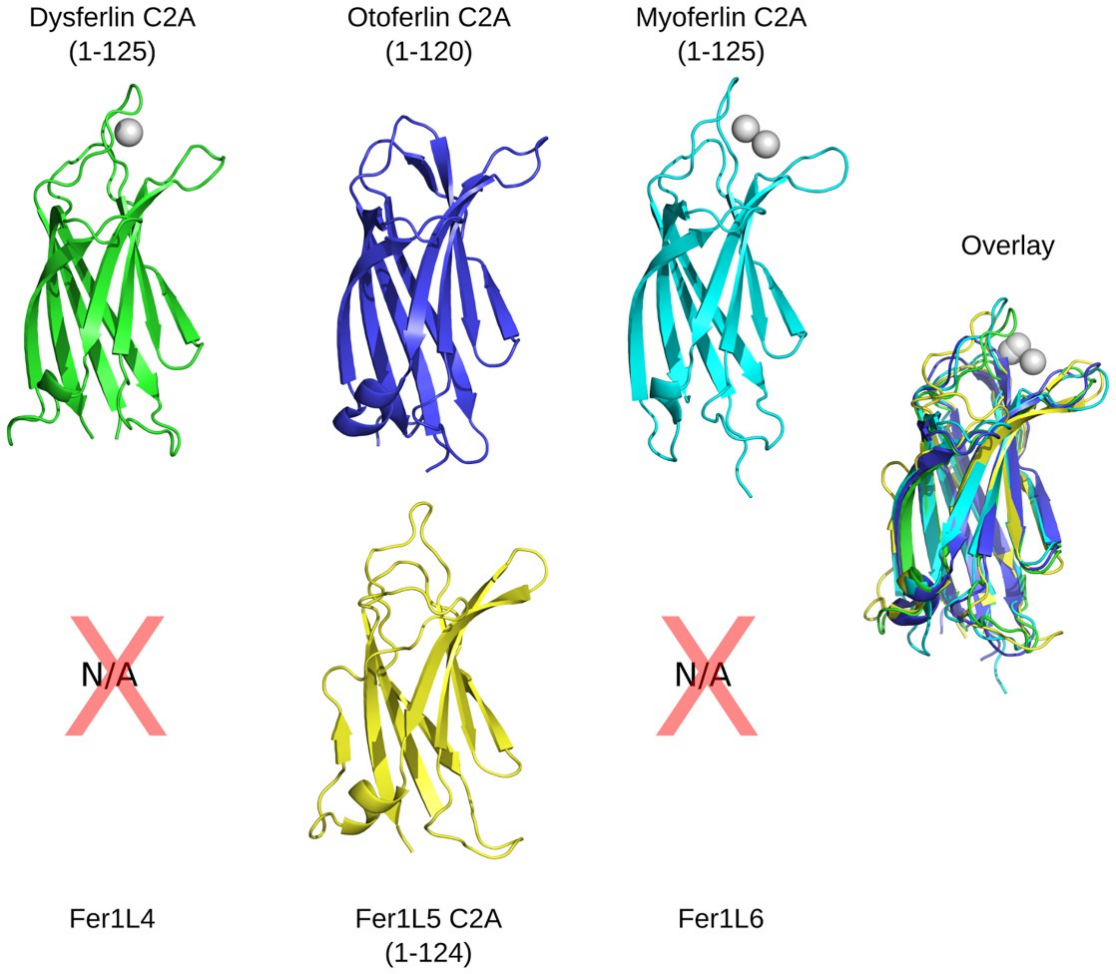
C2A structures and model of C2A from Fer1L5. Dysferlin C2A (green, 4IHB), otoferlin C2A (blue, 3L9B), myoferlin C2A (cyan, 6EEL), and Fer1L5 C2A (yellow). The gray spheres are the divalent cations from the crystal structures of dysferlin C2A (4IHB, chain E) and myoferlin C2A (6EEL). The amino acid boundaries from each respective C2 domain are listed with the domain assignment. The superposition of all four C2A domains is labeled as ‘Overlay’. The ferlins without a C2A domain are listed as Not Applicable (N/A).

Four of the human ferlin proteins: dysferlin, otoferlin, myoferlin, and, Fer1L5 possess domains identified as C2A [37] (S1-S17 Figs and S1-S7 Tables in S1 File). Fer1L4 and Fer1L6 begin with C2B domains [37] (Fig 1). Most of the structural and functional data on ferlin domains has focused on the C2A domain because the boundaries were simpler to predict [56–59]. The first residue of ferlin proteins with C2A domains correlates to the N-terminal residue of C2A, while the C-terminal C2A domain residue can be predicted from its overall similarity to other C2 structures. Currently, there are high-resolution crystal structures and solution-state NMR spectroscopy structures for dysferlin C2A [56, 58], myoferlin C2A [57, 60], and otoferlin C2A [59]. Additionally, dysferlin [56, 58] and myoferlin C2A [57] have been solved with liganded divalent cations. Dysferlin (4IHB) and myoferlin (6EEL) C2A domains are similar in overall structure with a 1.91 Å RMSD between the two structures [57]. The C2A domain of otoferlin lacks loop 1 of its putative Ca^2+^-binding pocket, so it cannot coordinate Ca^2+^ [59]. Fer1L5 C2A is the only ferlin without experimental structural analysis; however, our current model of Fer1L5 provides predictive power to infer its properties (Fig 3). Fer1L5 only has one acidic residue (Asp-69) out of six acidic residues in the Ca^2+^ binding region (CBR) typically used to coordinate Ca^2+^. Therefore, we predict that the human Fer1L5 C2A domain does not possess the capacity to bind Ca^2+^. *β*-strand 3 of the Fer1L5 C2A model has a poly-basic region similar to *β*-strand 3 of dysferlin C2A (Fig 3); therefore, it may possess the ability to bind to negatively charged phospholipid surfaces [61]. Additionally, loop 1 of Fer1L5 may bind phospholipid membranes via its hydrophobic residues, and loop 3 has several basic residues that may direct the coordination of negatively charged phospholipids (Fig 3).

### Analysis of ferlin C2A-C2B linkers

The linker between the C2A and C2B domains (A-B linker) is the longest linker sequence modeled in the human ferlin molecules. Our models predict this region to be mostly unstructured due to a high fraction of proline and glycine residues. For example, the A-B linker sequence in dysferlin is 93 amino acids in length and is 22% proline. The A-B linker sequence from myoferlin is 73 amino acids in length and is 13.7% proline and 13.7% glycine. Otoferlin has the longest A-B linker sequence in the ferlin family with 132 amino acids but possesses a lower fraction of proline and glycine, 8.3% and 9.1% respectively. Fer1L5 has the shortest A-B linker of the human ferlin family, 42 amino acids, and a lower fraction of proline and glycine, 4.8% and 7.1%, respectively. Despite the prevalence of proline and glycine in the A-B linkers among all ferlins, our RoseTTAFold models predict two *α*-helices near the center of the chain. The two helices are oppositely charged, and they are modeled as an electrostatically-coupled pair. The N-terminal helix tends to be negatively charged, while the C-terminal helix in the A-B linker tends to be positively charged. The A-B linker in dysferlin possesses two pathological mutations; P132L and A170E [62]. P132L occurs within a proline-rich region of the linker near the C-terminus of the C2A domain. The A170E mutation occurs within the negatively charged helical region of the linker.

In dysferlin, there are 14 consensus phosphorylation sites predicted within the helices of the A-B linker [63]. Interestingly, the exon 5a insertion of dysferlin adds 31 residues to its A-B linker [64], and another nine potential phosphorylation sites. The prevalence of potential phosphorylation sites in the dysferlin A-B linker suggests that charge modification via serine/threonine phosphorylation may be a regulatory mechanism.

### Analysis of ferlin C2B domains

**Fig 4:**
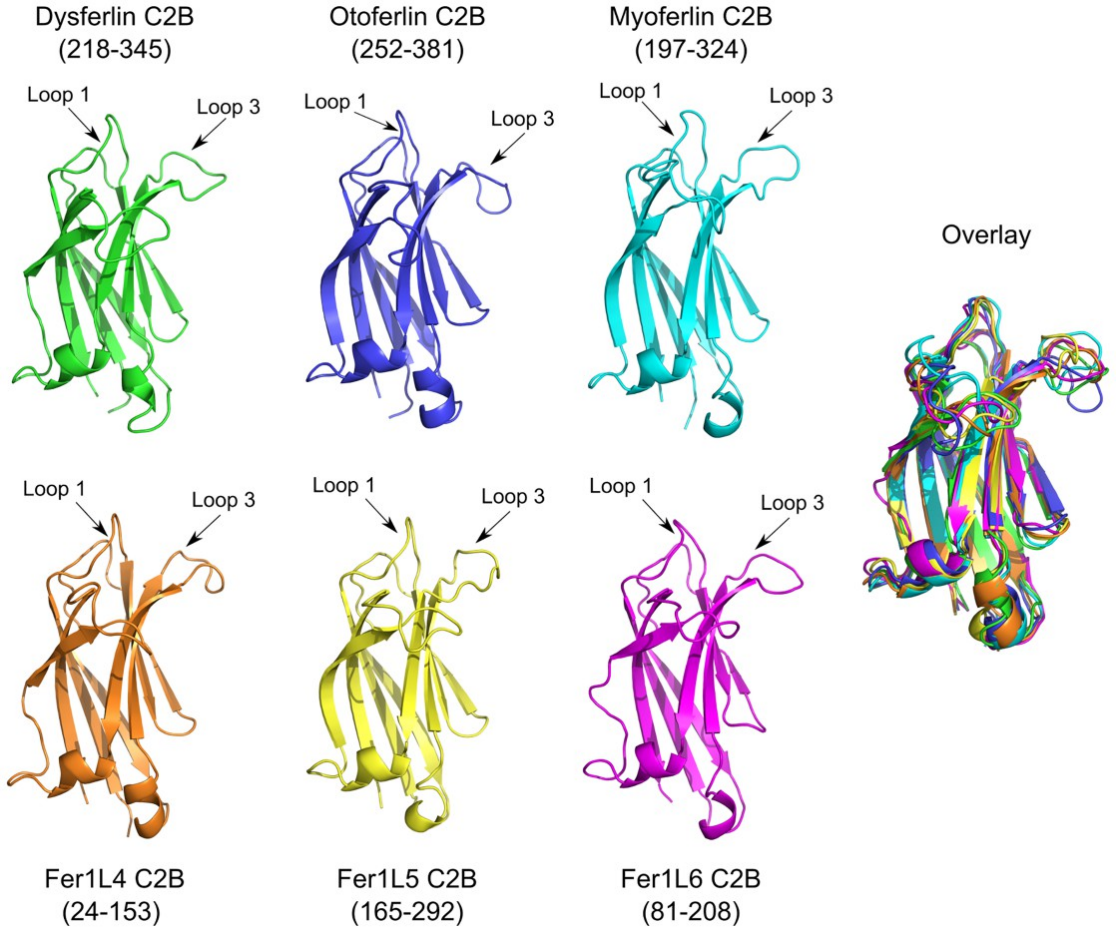
C2B models from dysferlin C2B (green), otoferlin C2B (blue), myoferlin C2B (cyan), Fer1L4 C2B (orange), Fer1L5 C2B (yellow), and Fer1L6 (magenta). Amino acid ranges for each domain are listed under the domain label. The superposition of all six C2B domains is labeled as ‘Overlay’.

The reported boundaries for the C2B domain of dysferlin are the most consistent among all of the previous C2B domain predictions [1, 31, 33, 34] (S1-S17 Figs and S1-S7 Tables in S1 File). The six ferlin C2B models from RoseTTAFold superimpose on each other with an RMSD less than 1.0 Å (Fig 4), suggesting a consistent fold between ferlin C2B domains. All human ferlin C2B domains closely resemble a prototypical Type-II C2 domain in both length and structure. The C2B domains of the ferlins generally lack the typical C2 domain Ca^2+^ binding motif (S1-S17 Figs and S1-S7 Tables in S1 File); however, they possess putative lipid-binding residues on loops 1 and 3 (Fig 4). Interestingly, the Type-2 ferlins (e.g. ferlins without a DysF domain: otoferlin, Fer1L4, and Fer1L6) possess a conserved histidine residue in loop 3 at a locus typically used for axial Ca^2+^ coordination in other divalent cation binding C2 domains (S1-S17 Figs and S1-S7 Tables in S1 File, **amino acid position labeled ‘X4’)**.

### Analysis of the ferlin FerI region

The FerI region is a unique ferlin primary sequence motif that is described as being tightly sandwiched between C2B and C2C [37]. Despite its conserved nature, no definitive function has yet been ascribed to FerI. Our modeling of all six human ferlin proteins shows that FerI is approximately 30 residues in length and consists mostly of unstructured loop (Table 1). Interestingly, the FerI loop, in the full-length ferlin models which possess a C2A domain (dysferlin, myoferlin, otoferlin, and Fer1L5), can fold within a space between the C2A, C2B, and C2C domains. Conserved acidic residues in FerI are therefore available to mediate intra-molecular interactions with other C2 domains. The potential of FerI to mediate connections between C2 domains is reflected in its overall pI. For example, in the ferlins that have a C2A domain, the pI of the FerI region tends to be more acidic (pI = 3.97–4.21), reflecting the increased likelihood that FerI can make salt-bridged or hydrogen-bonded interactions with residues in the C2A domain (Table 1). The FerI region of the ferlins without a C2A domain (Fer1L4 and Fer1L6) have calculated pI values that are less acidic (pI = 5.06–6.75) (Table 1).

**Table 1:** Primary sequence alignment of the FerI region of the six human ferlin proteins.

Ferlin	Length	FerI Span	Sequence	pI
Dysferlin	31	346–376	PGDEAPLERKDPSEDKEDIESNLLRPTGVAL--	**4.21**
Otoferlin	31	382–414	KGDNIKTPHKANETDEDDIEGNLLLPEGVPPER	**3.93**
Myoferlin	33	325–355	TGDEPPPERRDRDNDSDDVESNLLLPAGIAL--	**4.40**
Fer1L4	33	154–186	RGDLPPPMLPPAPGHCSDIEKNLLLPRGVPAER	6.75
Fer1L5	30	293–322	VGDQALIDQKLLY-GTDDTDIQIFKSAVVPI--	**3.97**
Fer1L6	32	209–240	KGDVLKTSPKTS-DTEEPIEKNLLIPNGFPLER	5.06

The pI values of the FerI primary sequences highlighted in bold are more acidic.

### Analysis of ferlin C2C domains

**Fig 5:**
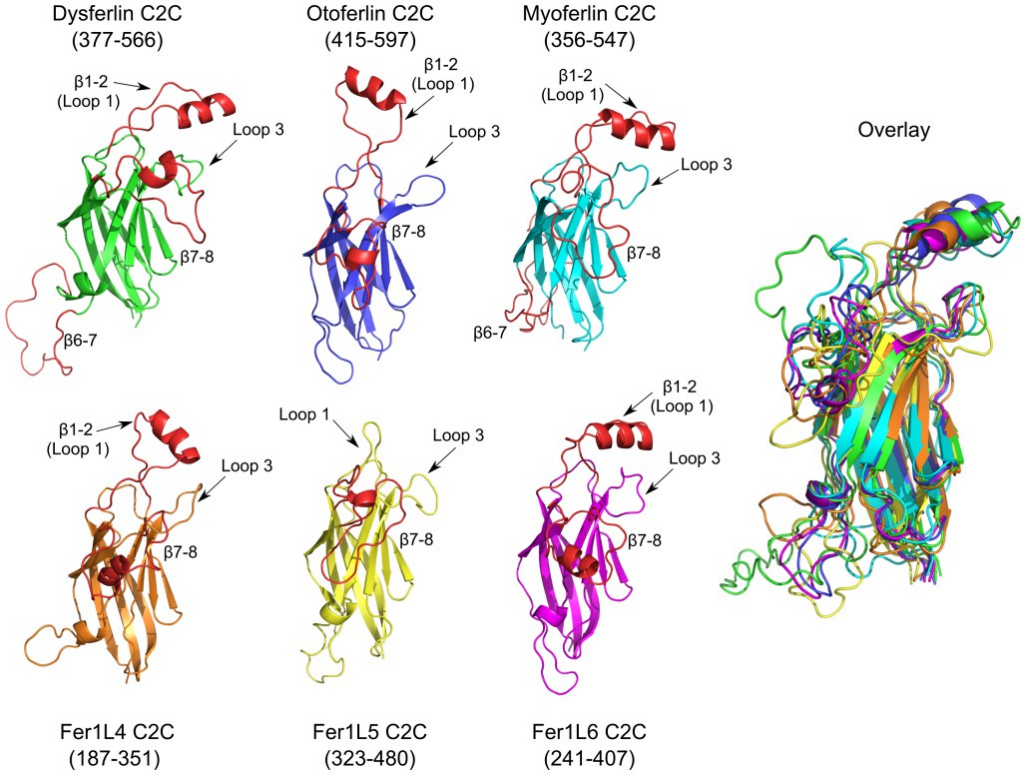
C2C models for dysferlin C2C (green), otoferlin C2C (blue), myoferlin C2C (cyan), Fer1L4 C2C (orange), Fer1L5 C2C(yellow), and Fer1L6 C2C (magenta). Amino acid ranges for each domain are listed under the domain label. The *β*1–2, *β*6–7, and *β*7–8 subdomains for C2C are labeled and shown in red. The superposition of all six C2C domains is labeled as ‘Overlay’.

The C2C domain is one of the most intricate C2 domains in the ferlin family, since it has more inter-*β*-strand subdomain insertions than any other ferlin C2 domain (Fig 5). The first subdomain of C2C occurs between *β*-strands 1 and 2 (*β*1–2), introducing a long, amphipathic *α*-helix within loop 1 of the C2 domain (**S1–S7 Tables and S1–S17 Figs in S1 File, labeled as ‘*α*1’**). The *β*1–2 subdomain of C2C is the only example of a loop 1 or *β*1–2 subdomain in the ferlin family. A loop 1 insertion is particularly notable as this loop typically participates in Ca^2+^-dependent phospholipid binding in many C2 domains. In fact, the *β*1–2 subdomain of C2C shares similarity to the loop 1 (CBR1) insertion observed in cytosolic phospholipase A2 [65]. We predict that C2C would possess a similar Ca^2+^ and phospholipid-binding activity as cytosolic phospholipase A2. In the Ca^2+^-dependent C2 domains, the domain itself contributes at least five of six total coordinating oxygen atoms to bind Ca^2+^; a phospholipid headgroup contributes the remaining coordinating oxygen. All six human ferlin C2C domains possess most of the requisite divalent cation coordination residues to bind Ca^2+^ (S1-S17 Figs and S1-S7 Tables in S1 File). Although the precise identification of the residues that could bind Ca^2+^ from the domain is not clear from modeling, the *β*1–2 subdomain could contribute acidic residues to complete the hexadentate Ca^2+^ coordination. Interestingly, five of the six ferlin C2C models are predicted to include the loop 1 subdomain; only Fer1L5 lacks this *β*1–2 subdomain (Fig 5).

The C2C domains of dysferlin and myoferlin possess an additional 30-amino acid subdomain between *β*-strands 6–7 (*β*6–7) (S1-S17 Figs and S1-S7 Tables in S1 File, labeled as ‘*α*2’). The overall structure of the *β*6–7 subdomain of C2C is uncertain; but, it may contain at least two *α*-helices. The exon 17 exclusion of dysferlin abbreviates the *β*6–7 subdomain to a single Val residue, making it a similar size to the other human ferlin C2C domains (S1-S17 Figs and S1-S7 Tables in S1 File) [64]. Interestingly, the exon 17 exclusion isoform of dysferlin is characteristic of the dysferlin transcripts present in blood tissue; yet it is effectively absent in muscle tissue [64]. The third subdomain of C2C occurs between *β*-strands 7–8 (*β*7–8), and it is approximately 20 residues in length across all human ferlins. There is no obvious sequence similarity or discernible function that can be inferred from our models or alignment of *β*7–8 (S1-S17 Figs and S1-S7 Tables in S1 File, **labeled as ‘*α*3’**).

### Analysis of ferlin C2-FerA domains

**Fig 6:**
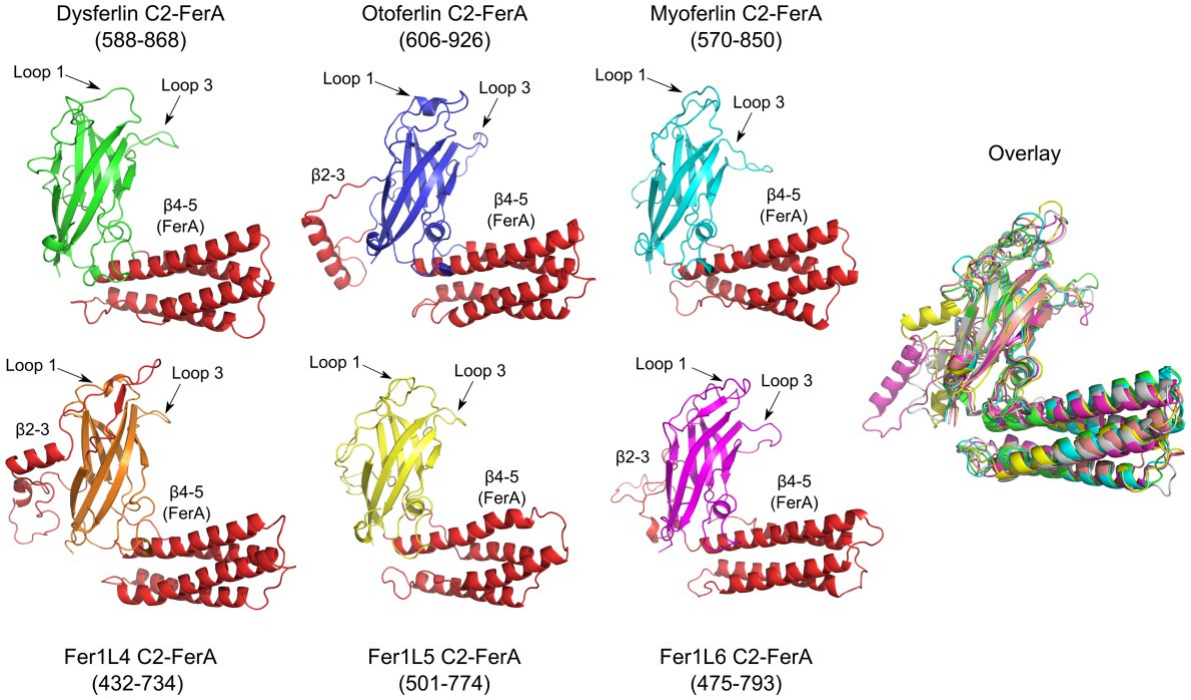
C2-FerA models for dysferlin C2-FerA (green), otoferlin C2-FerA (blue), myoferlin C2-FerA (cyan), Fer1L3 C2-FerA (orange), Fer1L5 C2-FerA (yellow), Fer1L6 C2-FerA (magenta). The FerA subdomain (*β*4–5) is shown as red *α*-helices, as is the *β*2–3 subdomain unique to Type-2 ferlins. The superposition of all six C2-FerA models is labeled as ‘Overlay’.

**Fig 7:**
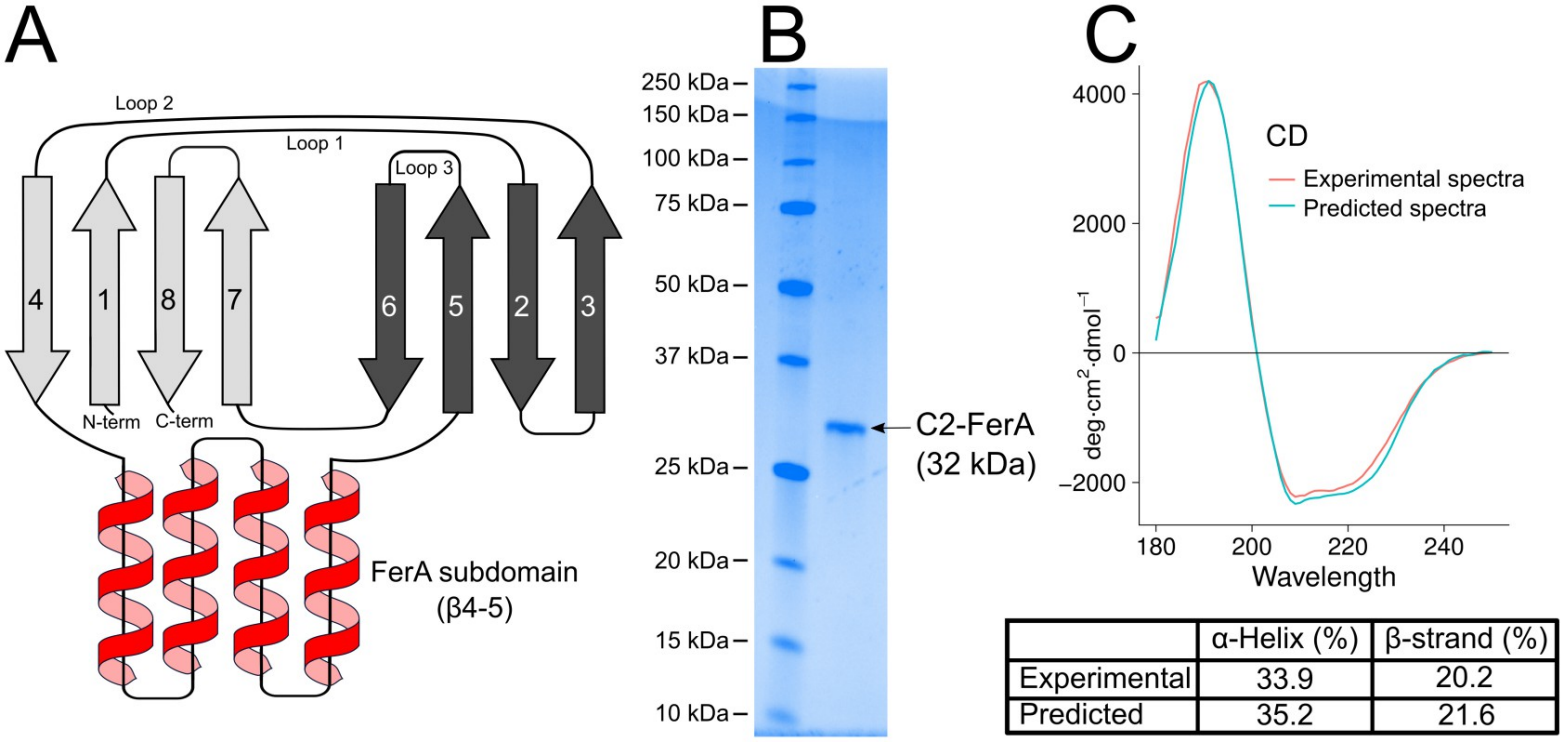
Dysferlin C2-FerA analysis. **A.** C2-FerA domain schematic showings the secondary structure connectivity and the large insertion of the *β*4–5 FerA subdomain. **B.** SDS-PAGE showing the purified dysferlin C2-FerA domain versus molecular weight size markers. **C.** Far-UV CD spectrum of purified dysferlin C2-FerA (red curve) and predicted C2-FerA spectrum derived from the model (blue curve). The inset table reports the secondary structure summary of the purified dysferlin C2-FerA domain (Experimental), and the dysferlin C2-FerA model (Predicted).

The FerA subdomain was initially discovered based on the predicted *α*-helical propensity of the dysferlin amino acid region located between C2C and inner DysF [66]. The isolated FerA subdomain has been shown to interact with phospholipids in a Ca^2+^-dependent manner [66]; however, the exact mechanism and biological function of FerA are still under study. Prior to the availability of RoseTTAFold and AlphaFold2, we observed tell-tale *β*-strands from secondary structure analysis on either side of the FerA four-helix bundle [45]. Using the predicted flanking *β*-strands, we could assemble a complete C2 domain with the FerA four-helix bundle modeled as a subdomain between *β*-strand 4 and *β*-strand 5 (*β*4–5). The new C2 domain has been independently modeled in our AlphaFold2 and RoseTTAFold ferlin analysis (Fig 6). We refer to this novel C2 domain as C2-FerA. C2-FerA was likely not discovered in the initial automated domain searches of the ferlins because the large four-helix bundle that interrupts the typical C2-like motif is nearly as large as a prototypical C2 domain (Fig 7A, S1-S17 Figs and S1-S7 Tables in S1 File). C2-FerA is the largest domain by size in 5 out of the 6 human ferlins (Fig 1). Interestingly, the Type-2 ferlins (Fig 1) include an additional subdomain in C2-FerA that occurs between *β*-strands 2–3 (*β*2–3) (S1-S17 Figs and S1-S7 Tables in S1 File). The *β*2–3 subdomain is uniformly negatively charged in all three Type-2 ferlins.

To validate our C2-FerA model, we cloned, expressed, and purified the human dysferlin C2-FerA domain using domain boundaries from our RoseTTAFold results (Fig 7B). The secondary structure of the purified domain was measured by far-UV circular dichroism (CD) spectroscopy [50]. The experimental CD spectrum was consistent with a domain possessing a mix of *α*-helix and *β*-sheet secondary structural elements (Fig 7C). We also predicted the CD spectra of our C2-FerA model and computed the contribution of its secondary structural elements [51]. Both the experimentally derived CD spectra and the computed CD spectra agreed exceptionally well (Fig 7C); thus, confirming our C2-FerA domain model and validating the previously uncharacterized C2-FerA domain.

### Analysis of ferlin DysF regions

**Fig 8:**
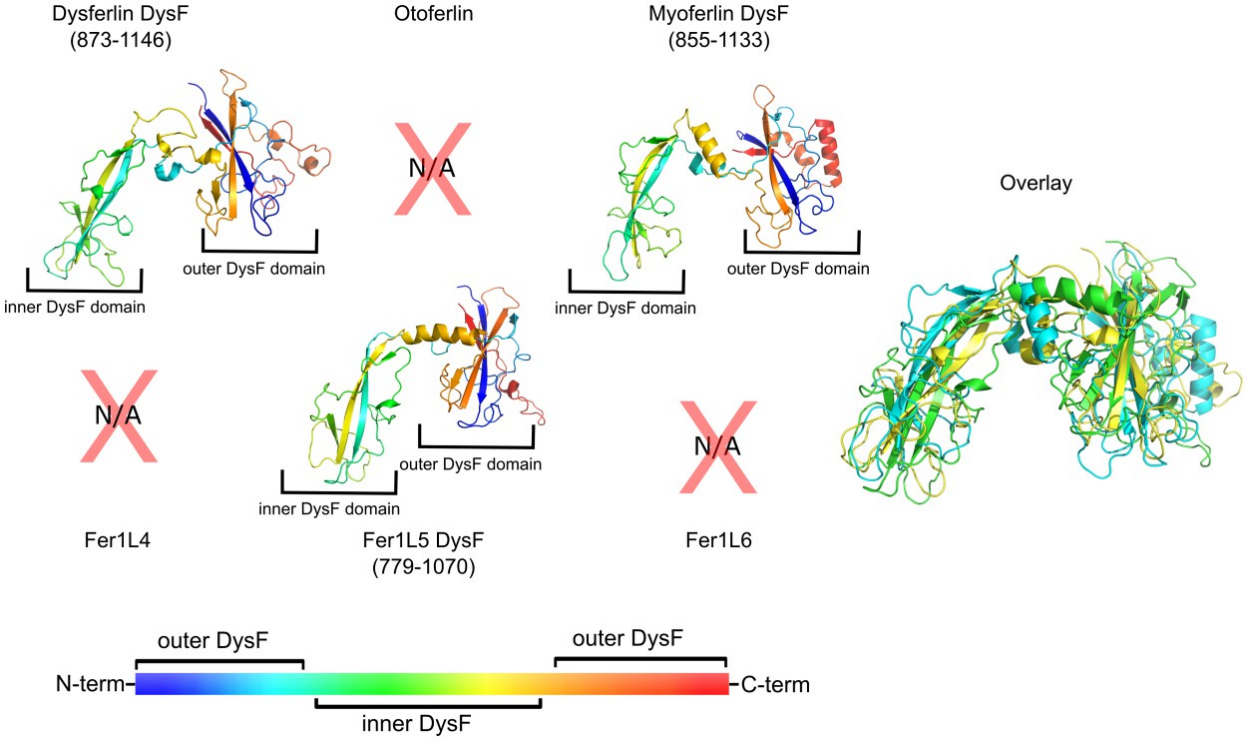
Models of the complete DysF region of the Type-1 ferlins; dysferlin, myoferlin, and Fer1L5. The inner DysF domain is positioned at the left of each model, whereas the outer domain is positioned on the right side. The models of the complete DysF region have been colored as a gradient from blue to red to highlight the crossover topology of the outer DysF domain’s fold. The color bar illustrates the progression of colors from N-terminus to C-terminus. The superposition of each of the Type-1 Ferlin DysF domains is labeled as ‘Overlay’.

The DysF region is unique to the Type-1 ferlins (Fig 1) and is composed of two DysF domains located near the center of the ferlin protein’s primary sequence [37]. The structures of the inner DysF domains of dysferlin and myoferlin were solved by X-ray crystallography and solution-state NMR spectroscopy, respectively [67, 68]. From primary sequence alignments, the inner DysF domain appears to be embedded within the sequence of the outer DysF domain (S1-S17 Figs and S1-S7 Tables in S1 File) [37]; however, it was unclear how to resolve the folding discontinuity using only the primary sequence. Our molecular modeling provides a possible solution to the complex topology of the DysF region (Fig 8). The inner DysF domain of dysferlin and myoferlin is indeed made up of two, contiguous anti-parallel *β*-strands, dominated by Trp-Arg *π*-cation interactions [67, 68]. The first *β*-strand of the outer DysF domain is located on the N-terminal side of the DysF region, while its second *β*-strand is located at the C-terminal end of the DysF region. The continuous inner DysF domain is embedded between the two halves of the outer DysF domain. The net result is two tandem DysF domains spaced apart by a single *α*-helix (Fig 8). The helix that separates the two DysF domains in dysferlin, myoferlin, and Fer1L5 is approximately 18-residues in length and possesses no outstanding biochemical characteristics, suggesting that it may serve as a spacer between DysF domains.

The physiological function of the DysF region of the Type-1 ferlins is unclear. Given the elongated structure of the ferlins, the DysF region is typically modeled at one focus of the major axis of the spheroid. The acentric orientation of the DysF domain in the context of the full-length protein may position DysF for productive phospholipid interaction [69]. There are examples of homologous DysF domains in *Saccharomyces cerevisiae* peroxisomal proteins Pex30p, Pex31p, and Pex32p where they are implicated in maintaining the number and size of peroxisomes [70, 71]. Additionally, the sporulation-specific protein 73 (SPO73) of *S. cerevisiae* is composed solely of a single DysF domain and is required for prospore membrane extension [72]. Interestingly, the DysF domain is only noted in one other human protein besides the Type-1 ferlins. The tectonin beta-propeller repeat-containing protein 1 (TECPR1) contains two DysF domains; however, the DysF domains are composed of continuous sequences and are not embedded as they are in the Type-1 ferlins. TECPR1 is involved in the fusion between autophagosomes and lysosomes, and it promotes the degradation of protein aggregates. Therefore, the DysF domain likely contributes to the membrane fusion/membrane binding activity of TECPR1 [73] and to the Type-1 ferlin proteins [69].

### Analysis of ferlin C2D domains

**Fig 9:**
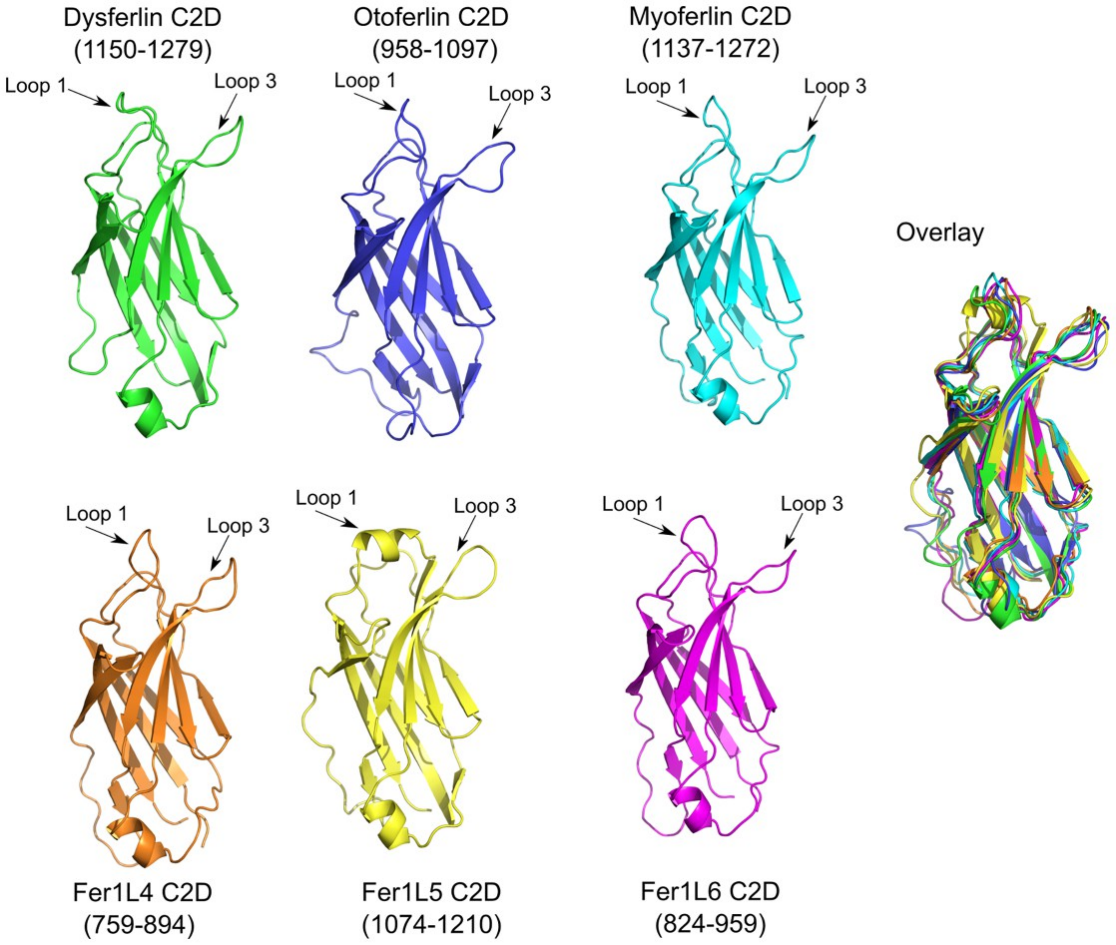
C2D models for dysferlin C2D (green), otoferlin C2D (blue), myoferlin C2D (cyan), Fer1L3 C2D (orange), Fer1L5 C2D (yellow), Fer1L6 C2D (magenta). The superposition of all six C2D models is labeled as ‘Overlay’.

C2D resembles a prototypical Type-II C2 domain with no discernible subdomains (Fig 9). Yet despite the straightforward 3D fold of C2D, there has been a wide variety of boundary estimates published for C2D [1, 31, 33, 34]. Five of the six C2D domains in the human ferlins possess all requisite residues typical of C2 domain Ca^2+^ coordination (S1-S17 Figs and S1-S7 Tables in S1 File). Indeed, the MIB (Metal Ion-Binding Site Prediction and Docking Server) server [74] predicts that Ca^2+^ can bind within the canonical divalent cation binding pocket of the ferlin C2D domains, except Fer1L5.

Loop 1 of the C2D domain of Fer1L5 is modeled as a C2C-like *α*-helix. Unlike the C2C domains in other ferlin proteins, the loop 1 *α*-helix in the C2D domain of Fer1L5 is not amphipathic; however, it does possess residues that could be used to bind phospholipid membranes (Fer1L5: Ile-1093 through Phe-1096). Loop 3 of Fer1L5 also possesses two bulky hydrophobic residues (Fer1L5: Phe-1157 and Trp-1158) with which to interact with the phospholipid membrane. In contrast to the *β*1–2 subdomain in C2C, the Fer1L5 C2D loop 1 helix does not possess residues that could participate in Ca^2+^ coordination, so Ca^2+^-dependent phospholipid interaction is unlikely (S1-S17 Figs and S1-S7 Tables in S1 File).

### Analysis of ferlin C2E domains

The Type-2 ferlins, otoferlin, Fer1L4, and Fer1L6, were originally thought to lack a C2E domain [37]. As a consequence of the missing C2 domain, an evolutionary analysis of the ferlin protein family required a large gap to align the seven C2 domains of the Type-1 ferlins with the five or six C2 domains of the Type-2 ferlins [37]. The missing C2 domain in the Type-2 ferlins caused a discrepancy in the sequential nomenclature of the ferlin C2 domains. To maintain a uniform nomenclature, all of the ferlin C2 domains were consecutively labeled C2A-C2F, except in Type-1 ferlins where the domain after C2D was labeled as ‘C2DE’, to reflect its location in the protein relative to its flanking C2 domains [37]. However, it is now clear from the RoseTTAFold and AlphaFold2 models that C2E is indeed present in all ferlins, including the Type-2 ferlins (Fig 1). In light of the discovery of the C2E domain in Type-2 ferlins, we recommend a return to the consecutive C2A-C2G nomenclature for all ferlins, with the exception of Fer1L4 and Fer1L6 that lack a C2A domain (Fig 1).

**Fig 10:**
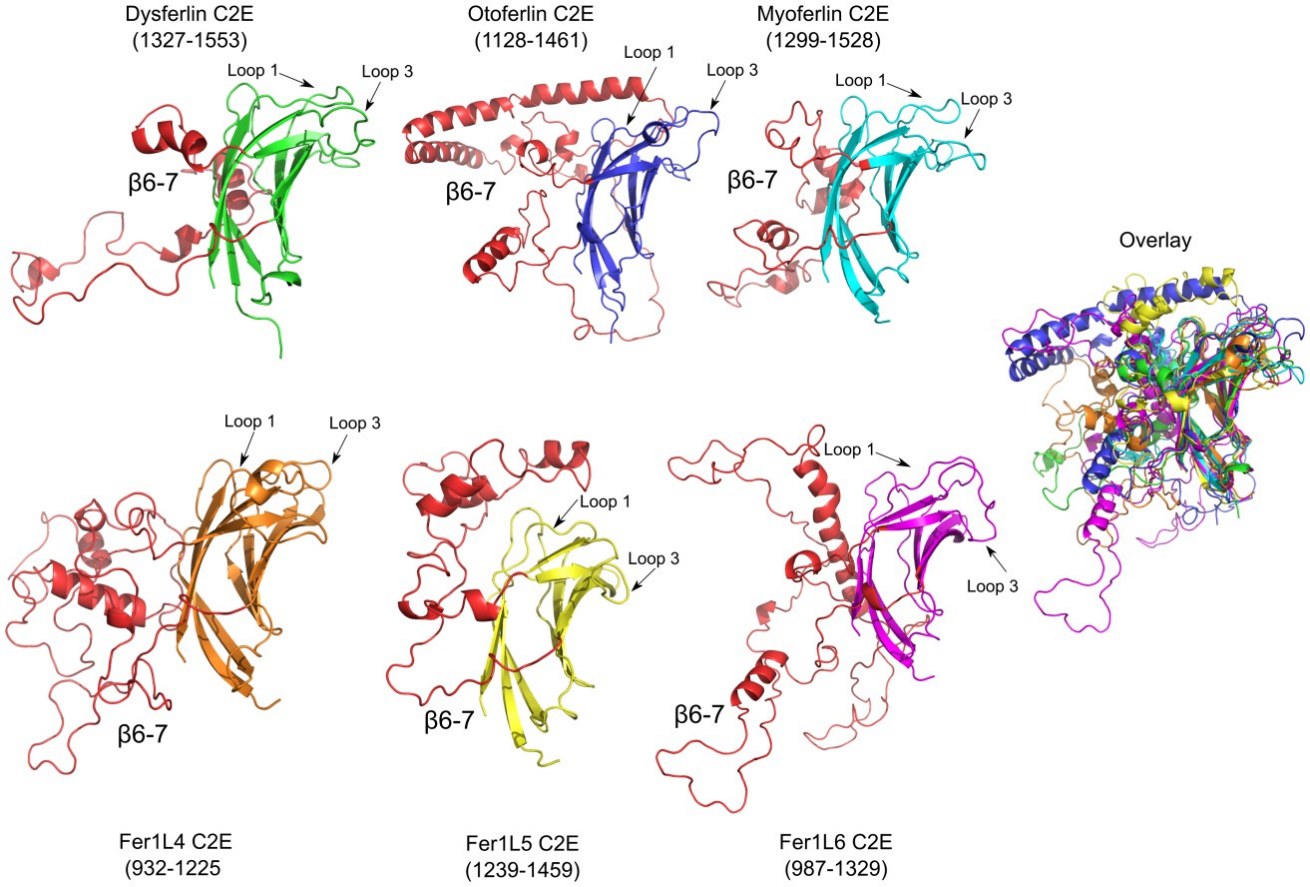
C2E models for dysferlin C2E (green), otoferlin C2E (blue), myoferlin C2E (cyan), Fer1L3 C2E (orange), Fer1L5 C2E (yellow), Fer1L6 C2E (magenta). The *β*6–7 subdomain of C2E is labeled and colored in red. The superposition of all six C2E models is labeled as ‘Overlay’.

The ferlin C2E domain contains a large subdomain between *β*-strand 6 and 7 (*β*6–7) (Fig 10, S1-S17 Figs and S1-S7 Tables in S1 File). The C2E *β*6–7 subdomain across the ferlin family is generally negatively charged, and it has been modeled as mostly *α*-helical. The overall length of C2E *β*6–7 varies widely between the ferlin proteins, and it is probably the reason C2E was overlooked in the original ferlin family analysis. The longest C2E *β*6–7 subdomains occur within otoferlin and Fer1L6, which are 203 and 214 residues in length, making the subdomains longer than the prototypical C2 domain. The *β*6–7 subdomains in dysferlin, myoferlin, Fer1L4, and Fer1L5 are 103, 106, 163, and 101 residues in length, respectively. Interestingly, the calpain cleavage site that produces a ‘synaptotagmin-like’ mini-dysferlin_C72_ (C2F-C2G-TM fragment) occurs within the *β*6–7 subdomain of C2E, but only in the exon40a insertion of human dysferlin [75]. Proteolytic cleavage at that site with calpain would remove six *β*-strands from the C2E domain and leave two *β*-strands as remnants on the N-terminus of the mini-dysferlin_C72_ fragment.

It is possible that C2E binds Ca^2+^ in the divalent binding pocket; however, the requisite constellation of acidic residues that are normally used to coordinate Ca^2+^ is currently unclear (S1-S17 Figs and S1-S7 Tables in S1 File). In each of the ferlin C2E domains, there are conserved Arg residues within the divalent cation binding pocket. For example, dysferlin Arg-1410 (S1-S17 Figs and S1-S7 Tables in S1 File) may act in the same manner as was observed in the C2Av1 (C2A variant 1) where positively charged residues salt-bridge to the Ca^2+^ binding residues; thus preventing Ca^2+^ binding [56]. The only exception is Fer1L5 where a Trp residue is predicted at that locus.

### Analysis of ferlin C2F domains

**Fig 11:**
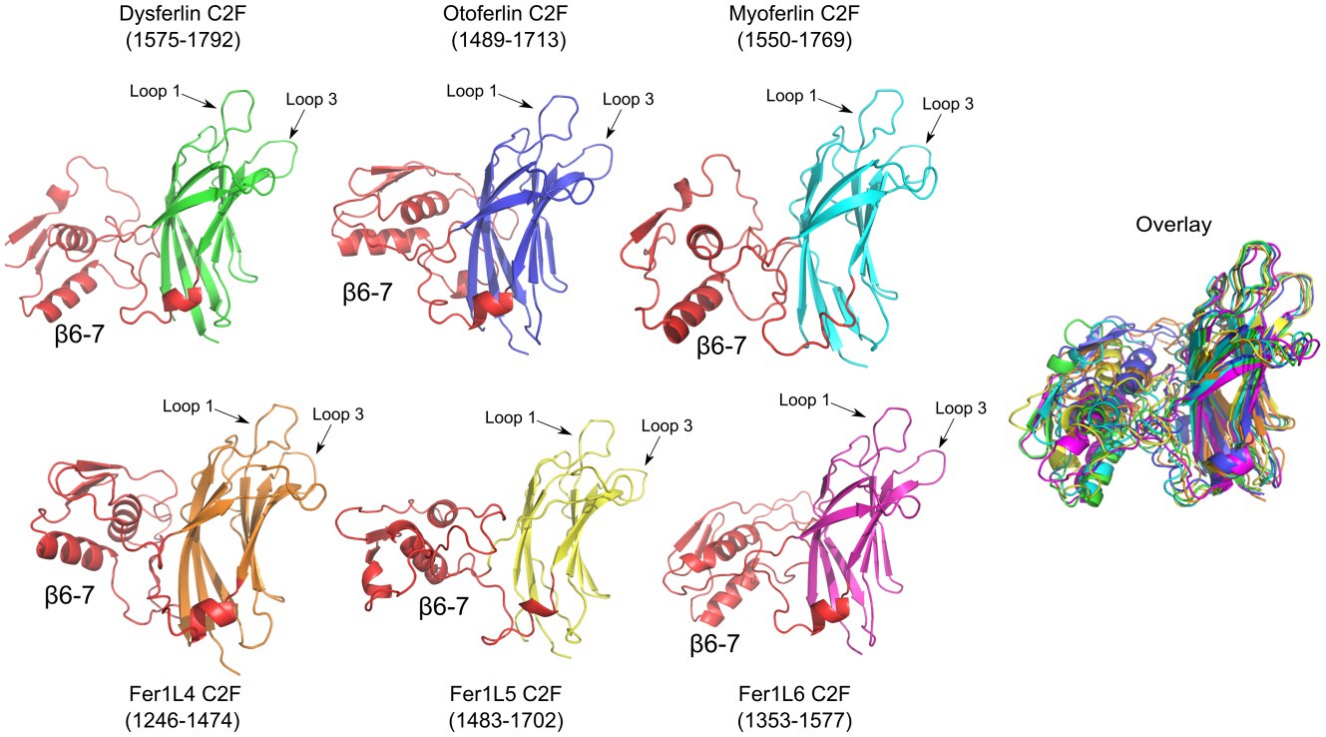
Dysferlin C2F (green), otoferlin C2F (blue), myoferlin C2F (cyan), Fer1L4 C2F (orange), Fer1L5 C2F (yellow), Fer1L6 (magenta). The *β*6–7 subdomain of C2F is labeled and colored red. The overlay of each domain is to the right of the figure.

**Fig 12:**
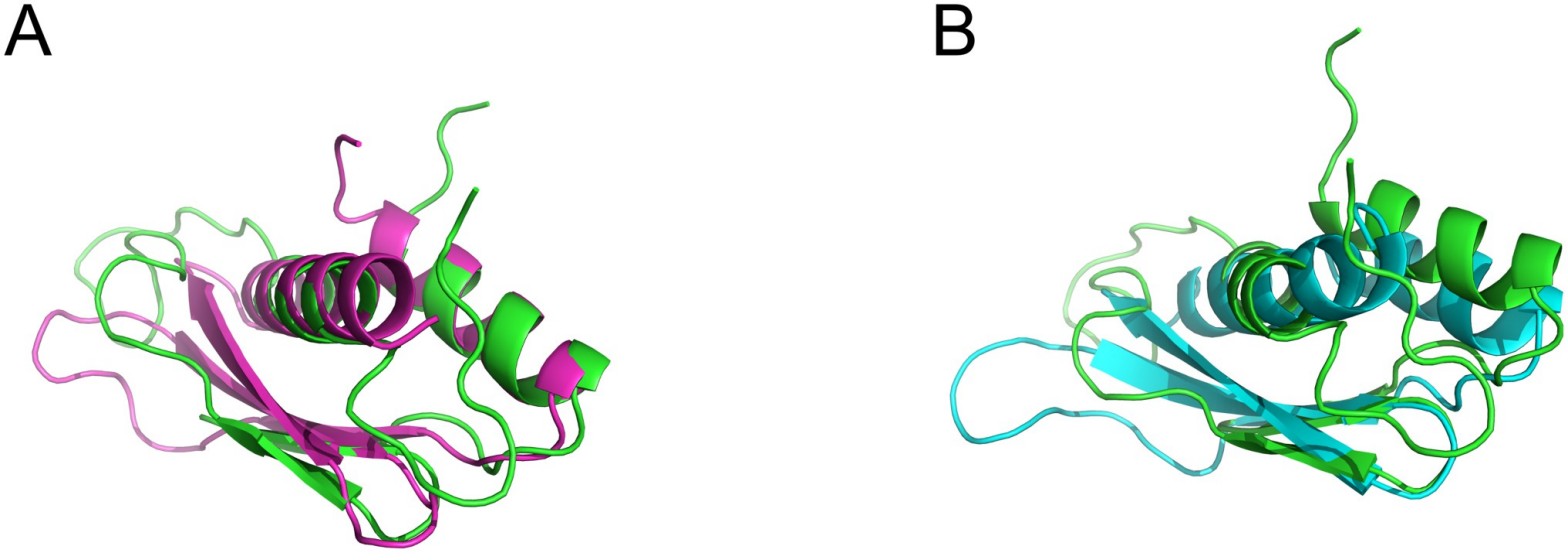
Dysferlin C2F *β*6–7 subdomain aligned with known dsRNA binding domains. **A.** Superimposed *β*6–7 subdomain of Dysferlin C2F (green) and RNA binding domain of Staufen (1STU) in cyan; RMSD = 2.7 Å. **B.** Superimposed *β*6–7 subdomain of Dysferlin C2F (green) and dsRNA binding domain of TARBP2 (4WYQ) in magenta; RMSD = 0.86 Å.

The C2F domain also resembles a prototypical C2 domain (Fig 11), with the exception of a unique subdomain between *β*-strands 6 and 7 (S1-S17 Figs and S1-S7 Tables in S1 File). Our ferlin models indicate that each C2F domain possesses all the conserved acidic amino acids to bind Ca^2+^, and it likely interacts with phospholipid membranes similar to dysferlin C2A (S1-S17 Figs and S1-S7 Tables in S1 File) [76]. The ∼ 60 amino acid C2F *β*6–7 subdomain adopts an *α*-*β*-*β*-*β*-*α* fold in which the two *α*-helices lie on one face of a three-stranded anti-parallel *β*-sheet. Interestingly, the C2F *β*6–7 subdomain shares structural similarity to a family of double-stranded RNA-binding (dsRNA) domains (Fig 12). For example, the dsRBD5 of the *Drosophila* Staufen protein [77] (1STU) superimposes with the human dysferlin *β*6–7 subdomain of C2F with an RMSD of 2.7Å (Fig 12A), and the RISC-loading complex subunit TARBP2 [78] (4WYQ) superimposes with an RMSD value of 0.86 Å (Fig 12B). It is unknown if any of the ferlins can bind dsRNA through the *β*6–7 subdomains of C2F, but the similarity to known dsRNA binding domains is intriguing. The residues that are used for dsRNA binding are not conserved in the ferlin C2F domains; however, otoferlin C2F conserves at least two of the four known residues that bind dsRNA.

### Analysis of ferlin C2G domains

**Fig 13:**
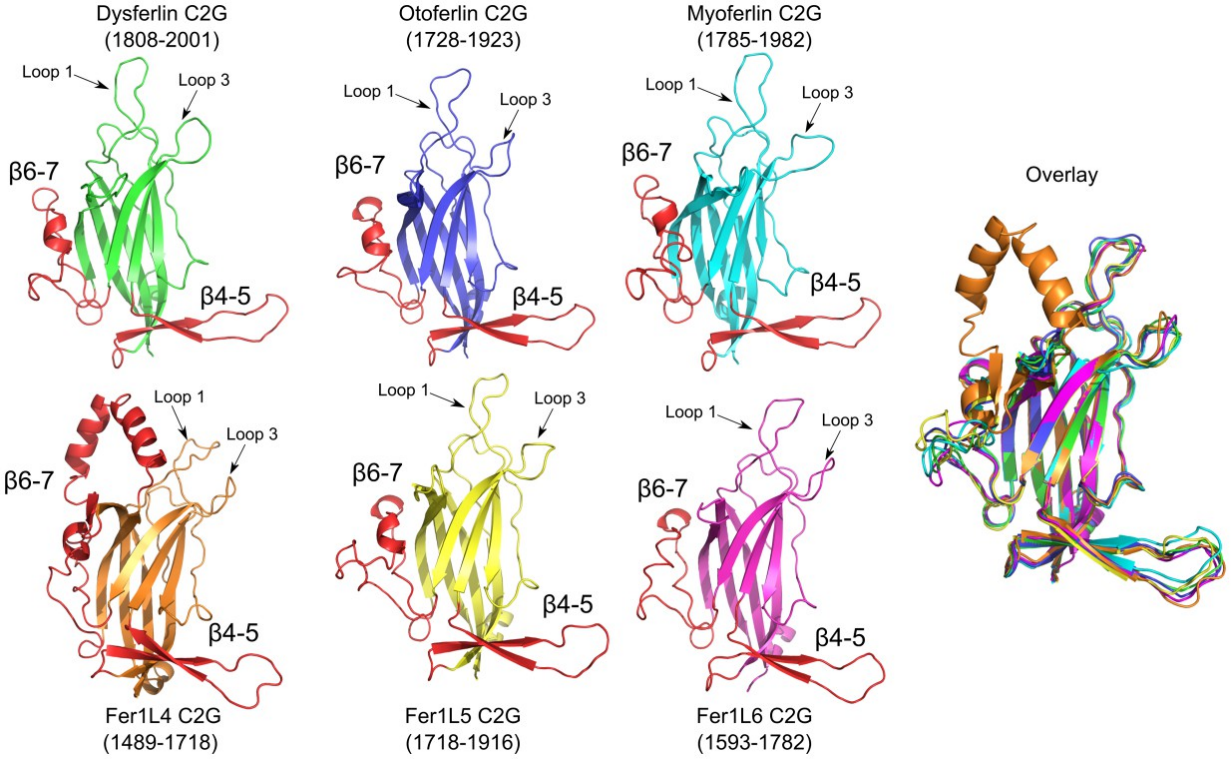
C2G models for dysferlin C2G (green), otoferlin C2G (blue), myoferlin C2G (cyan), Fer1L3 C2G (orange), Fer1L5 C2G (yellow), Fer1L6 C2G (magenta). The *β*4–5 and *β*6–7 subdomains of C2G are labeled and colored red. The superposition of all six C2G models is labeled as ‘Overlay’.

The C2G domain is the final C2 domain in the ferlin proteins prior to the transmembrane span (Fig 1). The models of each C2G domain from all human ferlins are predicted to be remarkably similar despite differences in sequence (Fig 13). In fact, each C2G model superimposes with a mean RMSD of 1.2 Å. The residues that coordinate Ca^2+^ are present within each ferlin C2G domain; therefore, we predict that all of the C2G domains should bind calcium ions (S1-S17 Figs and S1-S7 Tables in S1 File). There are two predicted subdomains in C2G. The first C2G subdomain has an antiparallel two-stranded *β*-sheet inserted between *β*-strands 4 and 5 (*β*4–5). The *β*4–5 subdomain of C2G gives the domain an overall ‘L’ shape. Due to the conservation of large hydrophobic residues at the tip of the C2G *β*4–5 subdomain throughout all ferlins (S1-S17 Figs and S1-S7 Tables in S1 File), it is possible that the C2G *β*4–5 subdomain assists in membrane binding. The second subdomain of C2G is located between *β*6–7 and is roughly 36 residues in length across all ferlins. The *β*6–7 subdomain is also well conserved among all ferlins except for Fer1L4 (S1-S17 Figs and S1-S7 Tables in S1 File). In Fer1L4, the *β*6–7 subdomain includes two long oppositely charged *α*-helices. The function of the *β*6–7 subdomain is unknown.

### Transmembrane (TM) spans and extracellular residues

Transmembrane (TM) spans were predicted from the full-length RoseTTAFold ferlin models and confirmed with the TMHMM server [79] (Table 2). The TM span for the human ferlins is predicted to be 23 residues in length except for Fer1L5, which is predicted to be 20 amino acids in length (Table 2). Each TM span and a conserved Pro residue at the C-terminus (S1-S17 Figs and S1-S7 Tables in S1 File). However, otoferlin is unusual in that its predicted TM span is shifted due to the substitution of a hydrophobic residue, typical of the other ferlins, for Lys-1968 immediately before the predicted helical insertion into the membrane (S1-S17 Figs and S1-S7 Tables in S1 File). The shift in otoferlin’s TM span prediction places the conserved proline (Pro-1987) within the membrane (Table 2). Interestingly, there is a clinically isolated mutation linked to auditory neuropathy at the conserved Pro residue in otoferlin (P1987R) [80] that likely disrupts the hydrophobic packing of otoferlin’s TM span. In dysferlin, there is a clinically-defined TM mutation A2066T that has been linked to LGMD-2B [81]. Ala-2066 occurs in the unstructured hinge residues between the TM and the extracellular helix and could interfere with the overall architecture of the TM segment. (S1-S17 Figs and S1-S7 Tables in S1 File).

**Table 2:** Predicted ferlin transmembrane span boundaries and extracellular residues.

Ferlin	Length	Transmembrane Span	Conserved Proline	Extracellular
Dysferlin	23	2045–2067	Pro–2068	2068–2080 (12)
Otoferlin	23	1969–1991	Pro–1987	1992–1997 (5)
Myoferlin	23	2026–2046	Pro–2049	2048–2061 (13)
Fer1L4	23	1758–1780	Pro–1781	1781–1794 (13)
Fer1L5	20	1962–1981	Pro–1983	1982–2047 (75)
Fer1L6	23	1824–1846	Pro–1846	1847–1857 (10)

The conserved ferlin Pro residues are listed. The numbers in parentheses indicate the number of predicted extracellular residues.

All ferlin TM helices appear to possess two short *α*-helical segments on either side of the membrane-spanning helix (S1-S17 Figs and S1-S7 Tables in S1 File). The short cytoplasmic and the extracellular helix are oriented parallel to the membrane, giving the TM helix an overall ‘Z’ shape (S1-S17 Figs and S1-S7 Tables in S1 File). To give the TM helix a ‘Z’ shape, ferlins possess several conserved hinging residues that break the helical propensity. On the N-terminal side of the ferlin TM span, there is a ‘WRRF’ sequence motif in dysferlin that forms the first flexible hinge (S1-S17 Figs and S1-S7 Tables in S1 File). These residues have previously been implicated in aggregating phosphatidylserine near the TM span [82]. The C-terminal short *α*-helix, potentially extracellular, is hinged by a conserved Pro residue that bends the TM span (S1-S17 Figs and S1-S7 Tables in S1 File, Table 2) [80].

## Discussion

Ferlins were first identified in 1980 as a gene (*fer-1*) essential for fertilization in *C. elegans* [83]. Later, the fer-1 protein was found to regulate Ca^2+^-dependent membrane fusion of membranous organelles during spermatogenesis [84]. A mammalian fer-1 ortholog was later designated as ‘*dys*-ferlin’ to reflect the deleterious role that mutations within the *DYSF* gene play in Limb-Girdle Muscular Dystrophy-2B (LGMD-2B) [85]. Five additional *fer-1* paralogs were subsequently annotated as ferlins in mammalian genomes. Phylogenetic analysis revealed that the ferlin family likely has an ancestral function as regulators of vesicle fusion and receptor trafficking [37, 86]. In the last several years, the role of ferlins has expanded to include vesicle fusion and plasma membrane repair [2, 9, 18, 87]. However, despite the indirect evidence implicating ferlins in membrane fusion, the mechanism is currently unknown.

Knowledge of the domain structure of the ferlins is essential to begin to understand its mechanism of action. Since at least 1998, [29] researchers have known that dysferlin possesses C2 domains; however, there was not a consensus on the number of C2 domains, the location of those domains within the primary sequence or the type/topology of each C2 domain [31, 33, 34]. Our analysis has described ferlin proteins as an amalgam of protein domains, and we show that ferlins possess up to eight C2 domains with Type-II topology. Additionally, our full-length ferlin models indicate an extended overall structure dominated by super-tertiary interactions. Although the full-length ferlin models lack confidence in the absolute domain positioning, they suggest the assemblage of ferlin C2 and DysF domains could function in a cooperative manner, which was previously unexpected.

The first domain in the ferlin protein family, C2A, resembles other known C2 domains in both size and topology, which made the boundary determination relatively straightforward. Additionally, the longest linker in the ferlin family is between the C2A and C2B domains creating a clear separation between predicted secondary structure elements (Fig 1). Previous analysis of dysferlin and myoferlin C2A shows that both C2A domains are capable of Ca^2+^-dependent membrane binding [56–58, 60]. The other ferlins either lack a C2A domain (Fer1L4 and Fer1L6) or, as is the case of the C2A domain of otoferlin, its Ca^2+^ binding activity has been abrogated by the lack of loop 1. [59]. Similarly, C2B is predicted to be a prototypical C2 domain in terms of size and topology (S1-S17 Figs and S1-S7 Tables in S1 File) and prior domain boundary predictions were remarkably consistent [1, 31, 33, 34]). C2B does not possess the requisite Ca^2+^-binding residues typical of other C2 domains; therefore, we conclude that C2B cannot bind Ca^2+^ in a manner similar to dysferlin or myoferlin C2A domains (S1-S17 Figs and S1-S7 Tables in S1 File). From the modeling of FerI in the various ferlin models, FerI domain seems to link C2A, C2B, and C2C via salt-bridged and H-bonded interactions. We speculate that the function of FerI is to limit the extensibility of the N-terminal ferlin domains. The C2C domain is the first domain in the ferlins that deviates significantly from the known structures of C2 domains. The C2C domain possesses the only *β*1–2 subdomain of all the ferlin C2 domains. A helical insertion with the *β*1–2 loop in the C2C domain is reminiscent of a similar insertion in phospholipase A2 [88]. In phospholipase A2, the larger amphipathic helix augments the surface area that can be used for Ca^2+^-dependent phospholipid interaction of the domain; a similar interaction could occur with C2C of the ferlins. C2-FerA folds like a C2 domain, but its activity seems to be focused on its unique *β*4–5 subdomain. There are no obvious lipid-binding residues on loops 1 or 3 of the C2 portion of C2-FerA, nor are there obvious Ca^2+^ binding residues. Therefore, the insertion of the four-helix bundle into the C2-FerA domain seems to be specialized for membrane engagement via the subdomain [57]. The *β*4–5 subdomain in isolation has been implicated in Ca^2+^-dependent membrane binding although the mechanism has not been described. From the model of C2-FerA, the domain likely binds phospholipids through hydrophobic interactions between the ‘opened’ FerA subdomain and a compatible membrane surface [66]. The DysF region is unique to the Type-1 ferlin proteins. The inner DysF domain is dominated by Trp-Arg interactions [67, 68] that likely augment the membrane interaction activity of the ferlins [69]. Similarly, the outer DysF models indicate a matching fold to inner DysF and the fold also appears to contain many Trp-Arg interactions. C2D seems to be a typical Ca^2+^-dependent phospholipid-binding C2 domain that is active on the C-terminal half of the ferlin proteins. C2E possesses the most extensive subdomain of all ferlins. The subdomain of C2E weaves throughout the full-length ferlin models and may facilitate C2 domain interactions in the globular state of the ferlins similar to the FerI model presented above (Fig 2, S1–S7 Figs and S1–S7 Tables in S1 File). Any Ca^2+^ binding activity will have to be assessed empirically since the models are not clear whether or not C2E can bind Ca^2+^ or phospholipid by itself. C2F is interesting in that it likely has all of the known Ca^2+^ and phospholipid-binding activity of a C2 domain, yet its subdomain may also bind dsRNA (Fig 12). Currently, it is unclear what role, if any, dsRNA may play in the function of ferlin proteins. C2G is the final C2 domain in the ferlin protein family, and it also possesses all the known Ca^2+^ and phospholipid-binding residues seen in dysferlin and myoferlin C2A domains. There are ten pathogenic mutations localized to dysferlin C2G, so understanding its 3D structure will be critical to understanding its function [62]. The predicted structure of the ferlin transmembrane span is also intriguing. There appears to be a general trend in the ferlins to maintain an overall ‘Z’ shape of the transmembrane helix (S1-S17 Figs and S1-S7 Tables in S1 File). Indeed, there are pathogenic mutations that may interrupt the structure and function of the TM.

**Fig 14:**
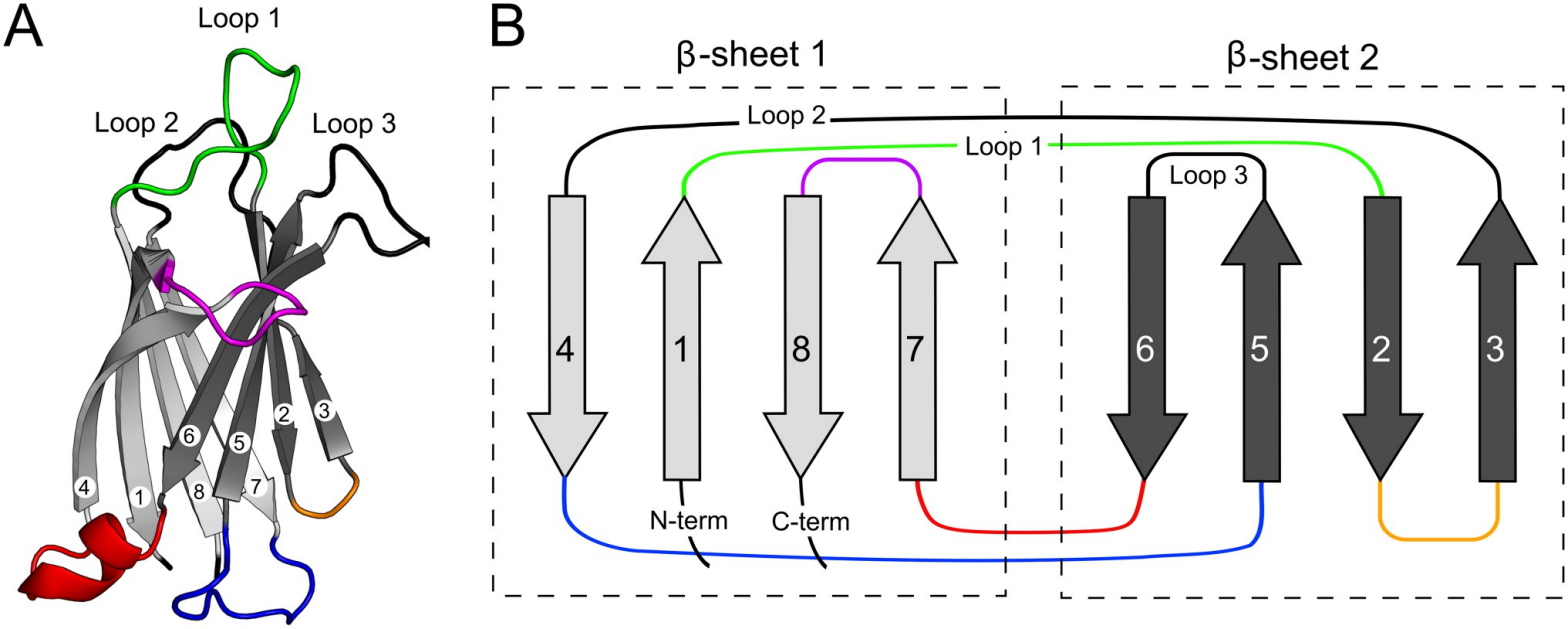
Schematic of Type-II C2 domain. **A:** Dysferlin C2A structure (4IHB) colored to highlighted the various insertions of subdomains. **B:**The schematic highlights the loops with embedded subdomains. The Type-2 C2 domain *β*-strand topology is shown as grey arrows. The colored loops: *β*1–2 (green, C2C), *β*2–3 (orange, C2-FerA), *β*4–5 (blue, C2-FerA, C2G), *β*6–7 (red, C2C, C2E, C2G, C2F), and *β*7–8 (purple, C2C) show where conserved subdomains are present.

Ferlins are part of the multiple-C2-domain-containing proteins (MC2Ds) superfamily classified as proteins containing two or more C2 domains [1, 89]. To our knowledge, ferlins are the only member of the MC2D superfamily that have C2 domains with extended loops forming conserved subdomains. Interestingly, C2A, C2B, and C2D do not possess extraneous subdomains, while C2C, C2-FerA, C2E, C2F, and C2G have elongated loops with unique characteristics (Fig 14). With the exception of C2C, loop extensions have been introduced in all of the inter-*β*-strand loops on the opposite side of the domain from the Ca^2+^ and phospholipid-binding pocket. The rationale for these modifications is not yet clear, but it may have to do with preserving the C2 domain’s overall shape and the C2 domain’s ability to interact with other domains and proteins [90].

RoseTTAFold- and AlphaFold2-derived models provide an excellent tool to decipher the domain structure of the ferlins, but further questions remain. For example, what are the ligand-binding residues of each domain, how do ferlins associate with membranes, how do pathogenic mutations affect the function of the protein, what is the function of the various subdomains, and do domain-domain intra-molecular interactions contribute to the overall activity? Understanding the structures and molecular mechanisms of large multi-functional ferlin proteins will be an important step toward understanding and treating ferlin-associated diseases such as Limb-Girdle Muscular Dystrophy type-2B (dysferlin), auditory neuropathy (otoferlin), and perhaps in the treatment of metastatic tumors (myoferlin).

## Supporting information

S1 File (PDF)

S1 Raw image (PDF)
