# Supplementary material for: Redefining the architecture of ferlin proteins: Insights into multi-domain protein structure and function
Source: PLoS One. 2022 Jul 28;17(7):e0270188. doi: 10.1371/journal.pone.0270188 (PMC9333456; doi:10.1371/journal.pone.0270188)
Supplement: S1 File — (PDF) [file pone.0270188.s001.pdf]

Redefining the architecture of ferlin proteins: insights into multi-domain protein structure and function

Matthew J. Dominguez[1], Jon J. McCord[1], R. Bryan Sutton[1,2]

1 Department of Cell Physiology and Molecular Biophysics, Texas Tech University Health Sciences Center, Lubbock, TX 79430, USA
2 Center for Membrane Protein Research, Texas Tech University Health Sciences Center, Lubbock, TX 79430, USA

*roger.b.sutton@ttuhsc.edu

Supporting information

Ferlin	C2A	C2B	C2C	C2-FerA	DysF	C2D	C2E	C2F	C2G	TM
Dysferlin	1-125	218-345	377-566	588-868	873-1146	1150-1279	1327-1553	1575-1792	1808-2001	2045-2067
Otoferlin	1-120	252-381	415-597	606-926	855-1133	958-1097	1128-1461	1489-1713	1728-1923	1969-1991
Myoferlin	1-125	197-324	356-547	570-850	n/a	137-1272	1299-1528	1550-1769	1785-1982	2026-2046
Fer1L4	n/a	24-153	187-351	432-734	n/a	759-894	932-1225	1246-1474	1489-1718	1758-1780
Fer1L5	1-124	165-292	323-480	501-774	779-1070	1074-1210	1239-1459	1483-1702	1718-1916	1962-1981
Fer1L6	n/a	81-208	241-407	475-793	n/a	824-959	987-1329	1353-1577	1593-1782	1824-1846

S1 Table. Ferlin domain boundary table. Summary of the ferlin C2 domain boundaries from the RoseTTAFold and AlphaFold2 models

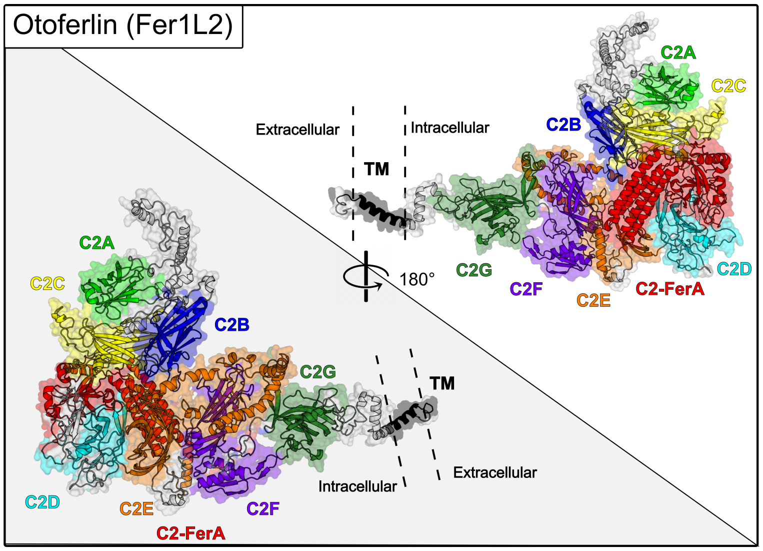

S1 Fig. Otoferlin RoseTTAFold model. The RoseTTAFold models that were used in this study were flexibly aligned using FATCAT [1]. Inconsistencies in the 3D models that were generated as a result of the elastic alignment process were repaired using PyMod [2]. Figures were rendered with PyMol and displayed as 180° views of the model. The various domains of otoferlin are shown as colored surfaces and similarly colored labels.

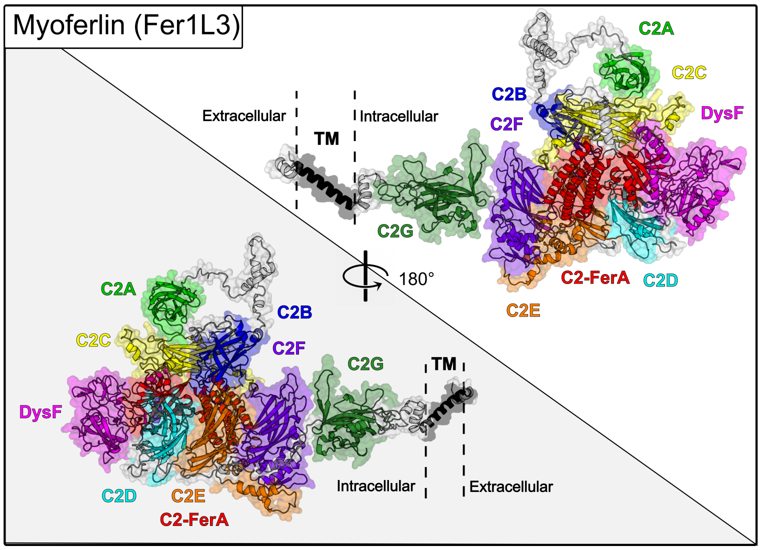

S2 Fig. Myoferlin RoseTTAFold model. The RoseTTAFold models that were used in this study were flexibly aligned using FATCAT [1]. Inconsistencies in the 3D models that were generated as a result of the elastic alignment process were repaired using PyMod [2]. Figures were rendered with PyMol and displayed as 180° views of the model. The various domains of myoferlin are shown as colored surfaces and similarly colored labels.

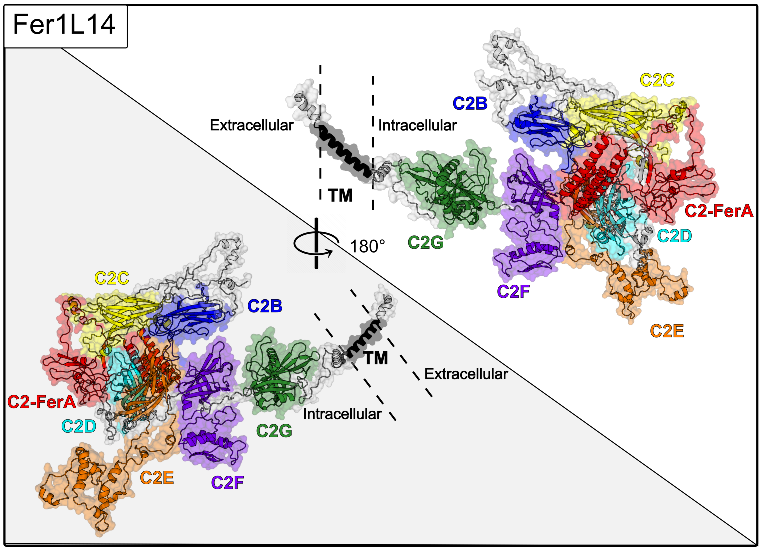

S3 Fig. Fer1L4 RoseTTAFold model. The RoseTTAFold models that were used in this study were flexibly aligned using FATCAT [1]. Inconsistencies in the 3D models that were generated as a result of the elastic alignment process were repaired using PyMod [2]. Figures were rendered with PyMol and displayed as 180° views of the model. The various domains of Fer1L4 are shown as colored surfaces and similarly colored labels.

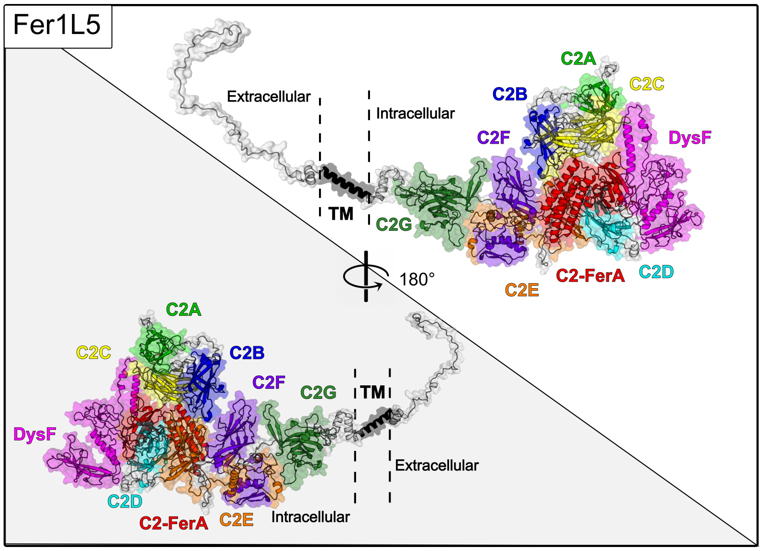

S4 Fig. Fer1L5 RoseTTAFold model. The RoseTTAFold models that were used in this study were flexibly aligned using FATCAT [1]. Inconsistencies in the 3D models that were generated as a result of the elastic alignment process were repaired using PyMod [2]. Figures were rendered with PyMol and displayed as 180° views of the model. The various domains of Fer1L5 are shown as colored surfaces and similarly colored labels.

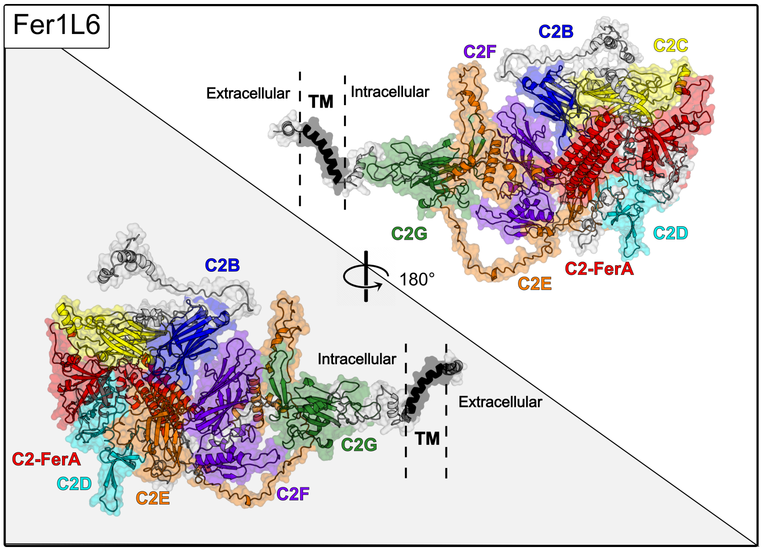

S5 Fig. Fer1L6 RoseTTAFold model. The RoseTTAFold models that were used in this study were flexibly aligned using FATCAT [1]. Inconsistencies in the 3D models that were generated as a result of the elastic alignment process were repaired using PyMod [2]. Figures were rendered with PyMol and displayed as 180° views of the model. The various domains of Fer1L6 are shown as colored surfaces and similarly colored labels.

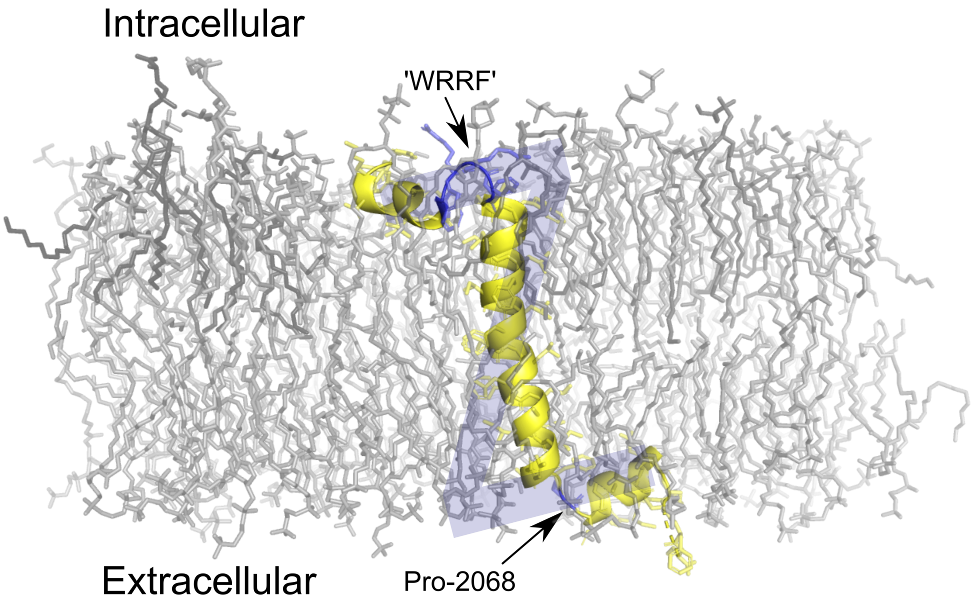

S6 Fig. Transmembrane span of dysferlin (yellow cartoon). The TM was simulated in an asymmetric membrane composed of 100% POPC on the extracellular side, and 80:10:10 POPC:POPS:POPG on the intracellular side (phospholipids shown as gray sticks). The system was assembled using CHARMM-GUI. The total simulation time was $\sim$ 1 millisecond for the phospholipid:protein:salt:water system. The 'WRRF' sequence is highlighted on the intracellular membrane, and the conserved Pro-2068 is highlighted on the extracellular membrane. The blue superimposed 'Z' shows the overall shape of the transmembrane span across all ferlins.

Dysferlin	C2A	C2B	C2C	C2-FerA	DysF	C2D	C2E	C2F	C2G
MolProbity Score	1.46	1.40	1.44	1.48	1.92	1.84	1.18	1.30	1.37
Clash Score	0.52	0.99	0	0.46	1.61	0.99	0	0.58	0.32
Ramachandran Favored (%)	89.52	93.40	87.12	90.04	92.08	92.31	90.62	96.02	93.08
Ramachandran Outliers (%)	0.95	0	3.07	3.03	2.08	0.96	2.60	0.57	0.63
QMEAN (Z-score)	-0.64	-0.87	-2.75	-2.21	-3.76	-1.65	-3.71	-1.13	-2.78

S2 Table. Model Scores for Dysferlin domains.

Otoferlin	C2A	C2B	C2C	C2-FerA	C2D	C2E	C2F	C2G
MolProbity Score	1.44	1.34	1.18	1.41	1.29	1.42	1.59	1.51
Clash Score	2.11	1.53	0.00	0.58	0.46	0.00	0.87	1.62
Ramachandran Favored (%)	92.86	93.07	90.21	93.17	93.39	90.39	90.12	90.91
Ramachandran Outliers (%)	0.00	1.98	3.50	0.72	3.31	3.16	0.58	1.82
QMEAN (Z-score)	-1.09	-1.07	-3.43	-1.66	-2.57	-3.34	-1.26	-2.54

S3 Table. Model Scores for Otoferlin domains.

Myoferlin	C2A	C2B	C2C	C2-FerA	DysF	C2D	C2E	C2F	C2G
MolProbity Score	1.39	1.70	1.80	1.63	1.67	1.22	1.50	1.51	1.46
Clash Score	0.00	1.00	1.02	0.68	0.69	0.95	0.55	0.29	0.95
Ramachandran Favored (%)	94.90	89.90	87.50	93.10	92.41	93.20	89.12	93.02	92.55
Ramachandran Outliers (%)	0.00	3.03	1.79	2.16	1.27	0.00	2.08	0.58	0.62
QMEAN (Z-score)	-1.93	-1.73	-1.99	-2.17	-3.25	-1.33	-2.94	-1.52	-2.22

S4 Table. Model Scores for Myoferlin domains.

Fer1L4	C2B	C2C	C2-FerA	C2D	C2E	C2F	C2G
MolProbity Score	1.70	1.60	1.52	1.57	1.52	1.96	1.33
Clash Score	1.46	1.57	0.62	0.93	0.22	0.58	0.54
Ramachandran Favored (%)	92.59	89.93	91.04	88.6	90.73	83.33	91.51
Ramachandran Outliers (%)	0.93	2.68	1.43	0.88	2.02	4.30	1.89
QMEAN (Z-score)	-1.71	-2.69	-1.46	-1.05	-3.56	-2.94	-1.97

S5 Table. Model Scores for Fer1L4 domains.

Fer1L5	C2A	C2B	C2C	C2-FerA	DysF	C2D	C2E	C2F	C2G
MolProbity Score	1.74	1.52	1.66	1.37	1.72	1.57	1.91	1.42	2.06
Clash Score	0.50	0.50	0.41	0.23	0.65	1.35	1.40	0.29	1.56
Ramachandran Favored (%)	89.32	92.31	81.20	92.41	89.11	90.91	87.43	89.67	89.51
Ramachandran Outliers (%)	1.94	0.00	2.56	0.84	2.42	2,73	1.57	1.09	1.23
QMEAN (Z-score)	-1.19	-1.84	-1.27	-2.10	-5.13	-2.34	-2.44	-1.21	-1.83

S6 Table. Model Scores for Fer1L5 domains.

Fer1L6	C2B	C2C	C2-FerA	C2D	C2E	C2F	C2G
MolProbity Score	1.44	1.28	1.54	0.86	1.30	1.39	1.73
Clash Score	0.52	0.78	0.61	0.00	0.19	0.29	1.65
Ramachandran Favored (%)	91.01	90.58	93.09	94.78	90.38	93.25	93.33
Ramachandran Outliers (%)	1.12	0.00	1.22	0.00	2.06	0.61	1.21
QMEAN (Z-score)	-2.18	-1.45	-1.38	-1.15	-2.65	-2.25	-2.44

S7 Table. Model Scores for Fer1L6 domains.

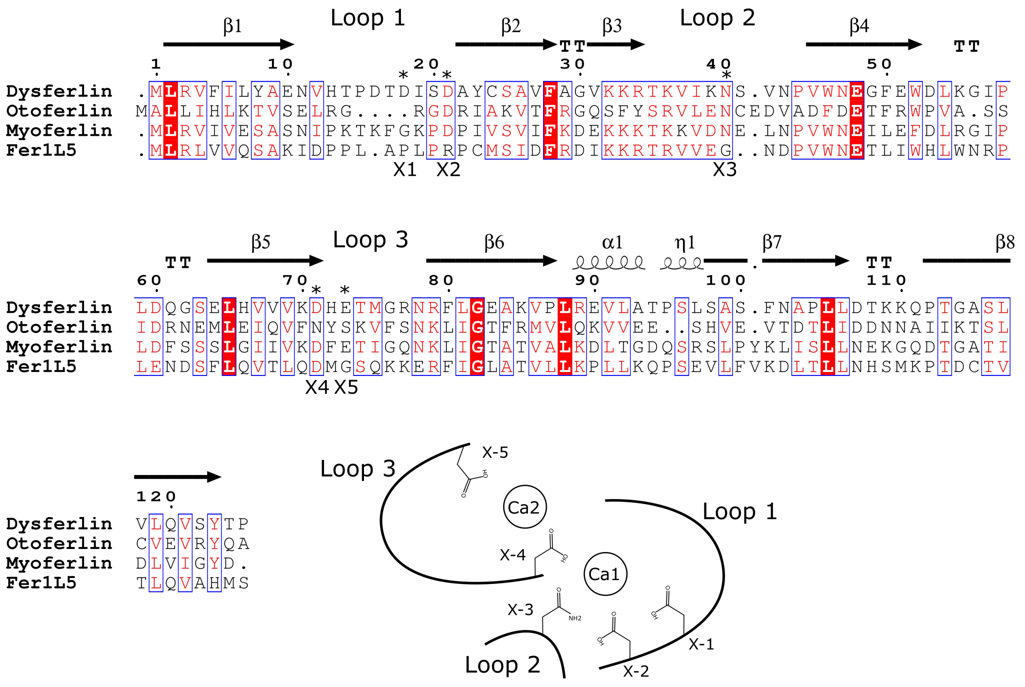

S7 Fig. Structure-based primary sequence alignment of the C2A domains of the ferlin family. Secondary structure assignments are based on the known and predicted structures of dysferlin as calculated by DSSP. The η symbol refers to a 3_{10}-helix. α-helices, 3_{10}-helices and π-helices are depicted as a generic helical cartoon. β-strands are rendered as arrows, strict β-turns as TT letters, and strict α-turns as TTT. Residues that are absolutely conserved between all C2A domains are highlighted in red. Conserved residues are boxed in blue. Numbers along the top of the alignment correspond to residue numbers in the dysferlin sequence. The mean evolutionary relatedness between the C2A domains of dysferlin, otoferlin, myoferlin, and Fer1L5 is 26.5% identity and 56.7% similarity. A schematic of the calcium ion binding region of the C2A domain is shown below the alignment. X-1, X-2, X-3, X-4, and X-5 demarcate the positions of the known Ca^{2+} binding residues in generic C2 domains.

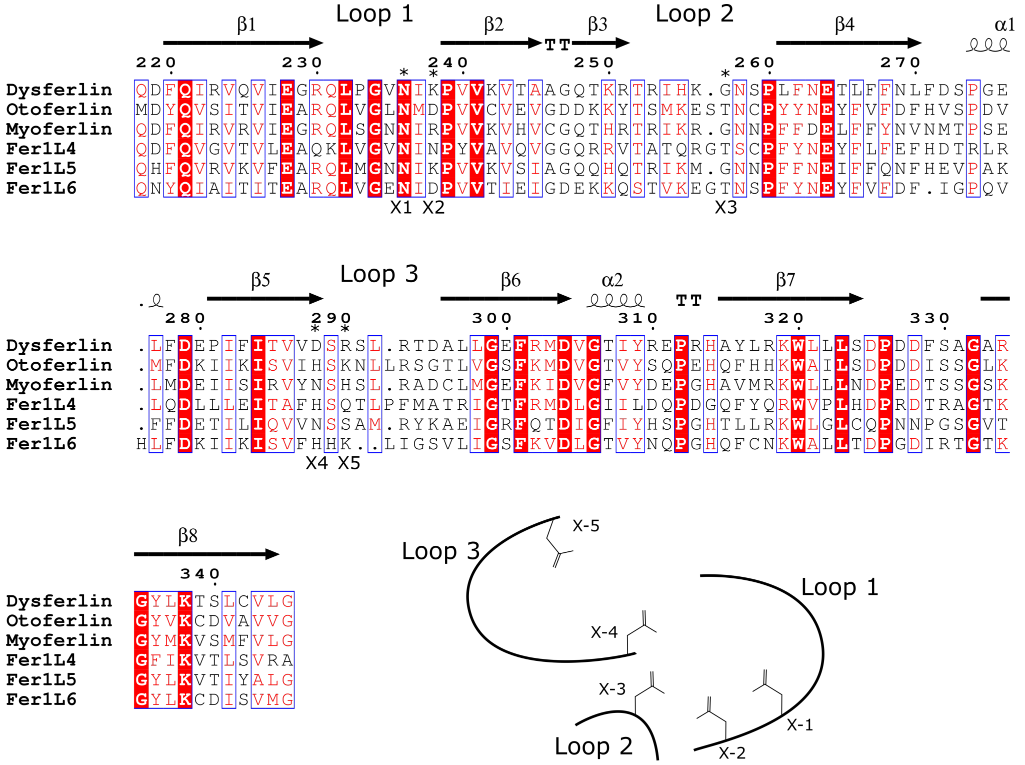

S8 Fig. Model-based primary sequence alignment of the C2B domains of the ferlin family. Secondary structure assignments are based on the predicted structures of dysferlin C2B as calculated by DSSP. The η symbol refers to a 3_{10}-helix. α-helices, 3_{10}-helices and π-helices are depicted as a generic helical cartoon. β-strands are rendered as arrows, strict β-turns as TT letters, and strict α-turns as TTT. Residues that are absolutely conserved between all C2B domains are highlighted in red. Conserved residues are boxed in blue. Numbers along the top of the alignment correspond to residue numbers in the human dysferlin sequence. The mean evolutionary relatedness between the C2B domains of dysferlin, otoferlin, myoferlin, Fer1L4, Fer1L5, and Fer1L6 is 44.4% mean identity and 63.8% mean sequence similarity across all six C2B domains. A schematic of the calcium ion binding region of the C2B domain is shown below the alignment. X-1, X-2, X-3, X-4, and X-5 demarcate the positions of the known Ca^{2+} binding residues in generic C2 domains. No coordinated calcium ions are shown in C2B as the binding residues are not uniformly present.

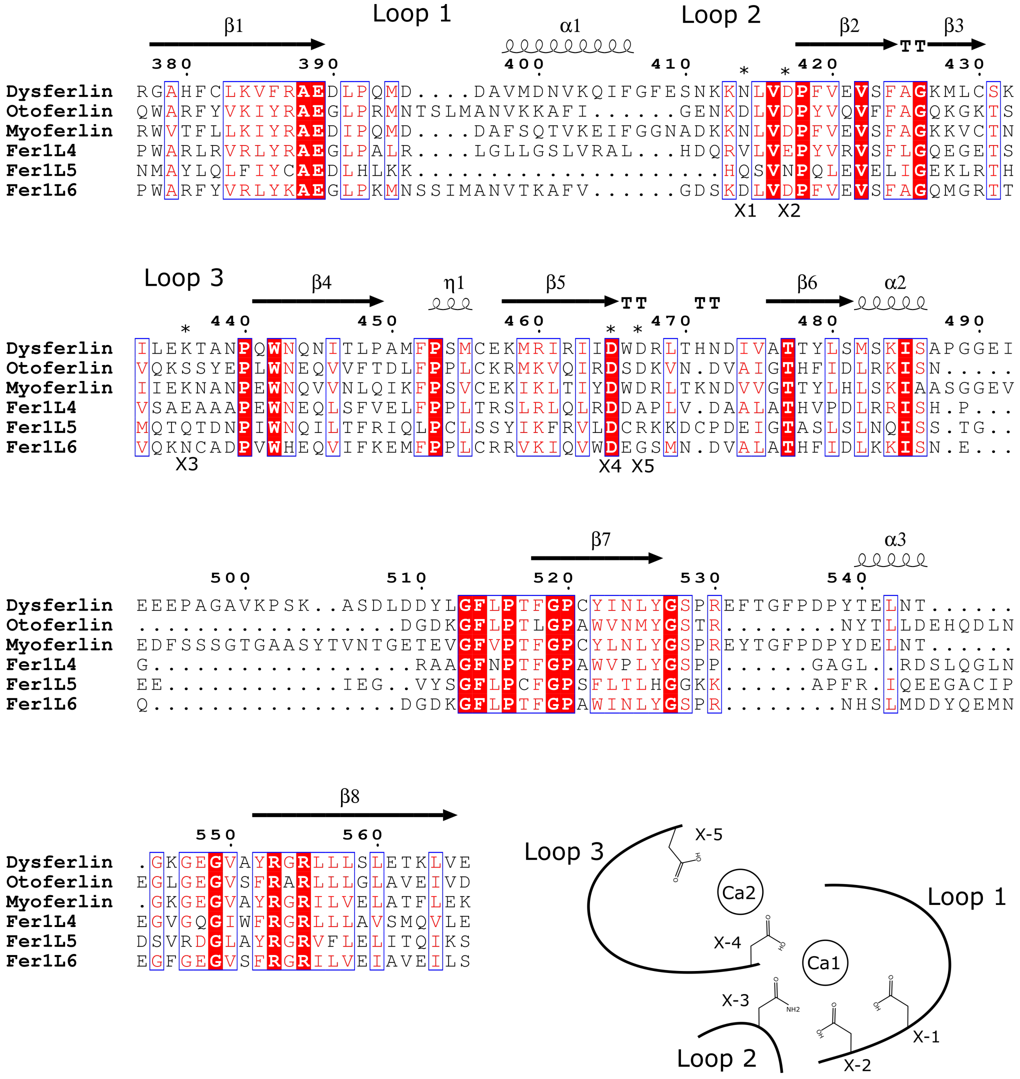

S9 Fig. Model-based primary sequence alignment of the C2C domains of the ferlin family. Secondary structure assignments are based on the predicted structures of dysferlin C2C as calculated by DSSP. The η symbol refers to a 3_{10}-helix. α-helices, 3_{10}-helices and π-helices are depicted as a generic helical cartoon. β-strands are rendered as arrows, strict β-turns as TT letters, and strict α-turns as TTT. Residues that are absolutely conserved between all C2C domains are highlighted in red. Conserved residues are boxed in blue. Numbers along the top of the alignment correspond to residue numbers in the human dysferlin C2C sequence. The mean evolutionary relatedness between the C2C domains of dysferlin, otoferlin, myoferlin, Fer1L4, Fer1L5, and Fer1L6 is 38.2% mean identity and 60.7% mean sequence similarity. A schematic of the calcium ion binding region of the C2C domain is shown below the alignment. X-1, X-2, X-3, X-4, and X-5 demarcate the positions of the known Ca^{2+} binding residues in generic C2 domains.

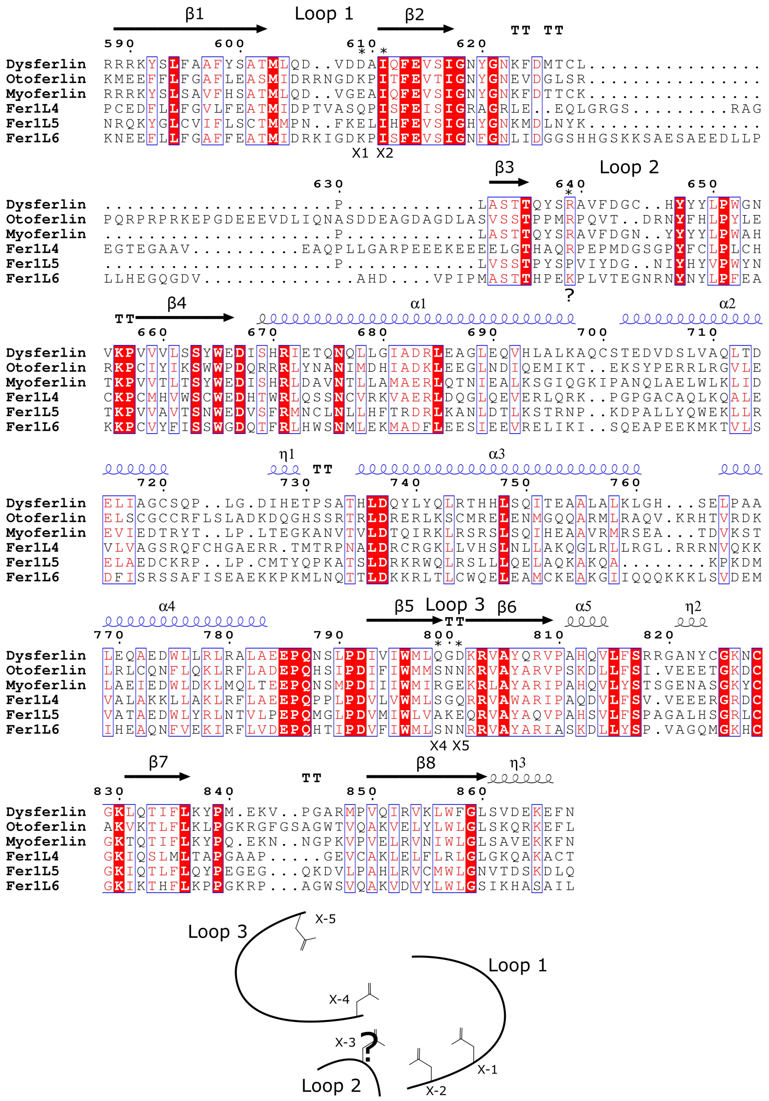

S10 Fig. Model-based primary sequence alignment of the C2-FerA domains of the ferlin family. Secondary structure assignments are based on the predicted structures of dysferlin C2-FerA as calculated by DSSP. The η symbol refers to a 3_{10}-helix. α-helices, 3_{10}-helices and π-helices are depicted as a generic helical cartoon. β-strands are rendered as arrows, strict β-turns as TT letters, and strict α-turns as TTT. Residues that are absolutely conserved between all C2-FerA domains are highlighted in red. Conserved residues are boxed in blue. Numbers along the top of the alignment correspond to residue numbers in the human dysferlin sequence. The blue helical cartoons demarcate the FerA helices between β strand-4 and β strand-5. The mean evolutionary relatedness between the C2-FerA domains of dysferlin, otoferlin, myoferlin, Fer1L4, Fer1L5, and Fer1L6 shows 34.9% mean identity and 55.3% mean sequence similarity for all six C2-FerA domains. A schematic of the calcium ion binding region of the C2-FerA domain is shown below the alignment. X-1, X-2, X-3, X-4, and X-5 demarcate the positions of the known Ca^{2+} binding residues in generic C2 domains. No coordinated calcium ions are shown in C2-FerA as the binding residues are not uniformly present.

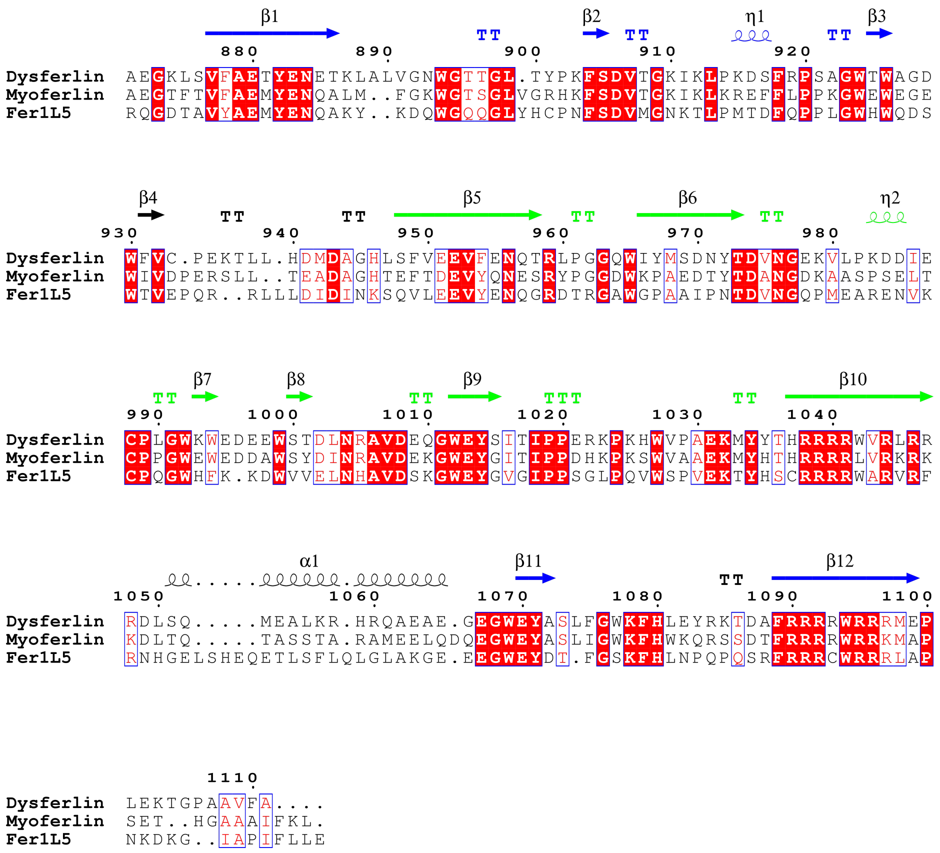

S11 Fig. Model-based primary sequence alignment of the DysF domains of the ferlin family. Secondary structure assignments are based on the known and predicted structures of dysferlin DysF as calculated by DSSP. The blue secondary structure is predicted to constitute the 'outer' DysF domain, while the green secondary structure demarcates the 'inner' DysF domain. The η symbol refers to a 3_{10}-helix. α-helices, 3_{10}-helices and π-helices are depicted as a generic helical cartoon. β-strands are rendered as arrows, strict β-turns as TT letters, and strict α-turns as TTT. Residues that are absolutely conserved between all DysF domains are highlighted in red. Conserved residues are boxed in blue. Numbers along the top of the alignment correspond to residue numbers in the human dysferlin sequence. The fold of the predicted dysferlin outer DysF domain superimposes with the known structure of the dysferlin inner DysF structure with an RMSD of 3.5 Å across all C-α residues. The mean evolutionary relatedness between the three DysF regions of the Type-I ferlins is 45.0% mean identity and 68.4% mean sequence similarity.

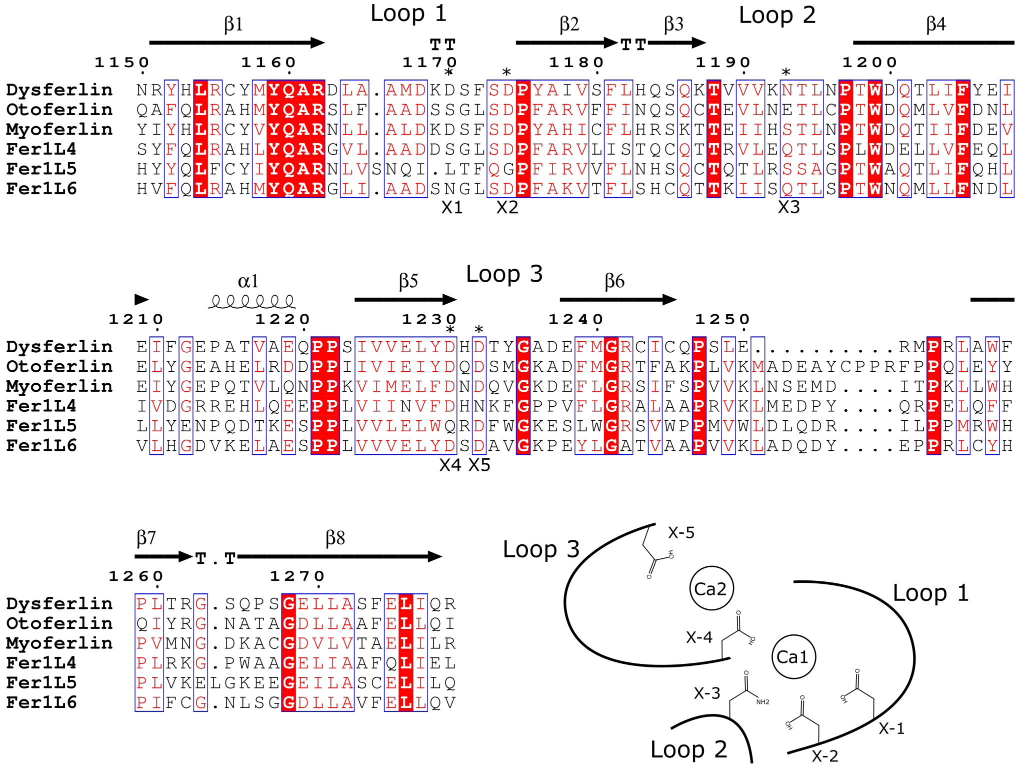

S12 Fig. Model-based primary sequence alignment of the C2D domains of the ferlin family. Secondary structure assignments are based on the predicted structures of dysferlin C2D as calculated by DSSP. α-helices are depicted as a generic helical cartoon. β-strands are rendered as arrows and strict β-turns as TT letters. Residues that are absolutely conserved between all C2D domains are highlighted in red. Conserved residues are boxed in blue. Numbers along the top of the alignment correspond to residue numbers in the human dysferlin sequence. The mean evolutionary relatedness between all six C2D domains shows 63.4% mean sequence similarity and 40.9% mean identity. A schematic of the calcium ion binding region of the C2D domain is shown below the alignment. X-1, X-2, X-3, X-4, and X-5 demarcate the positions of the known Ca^{2+} binding residues in generic C2 domains.

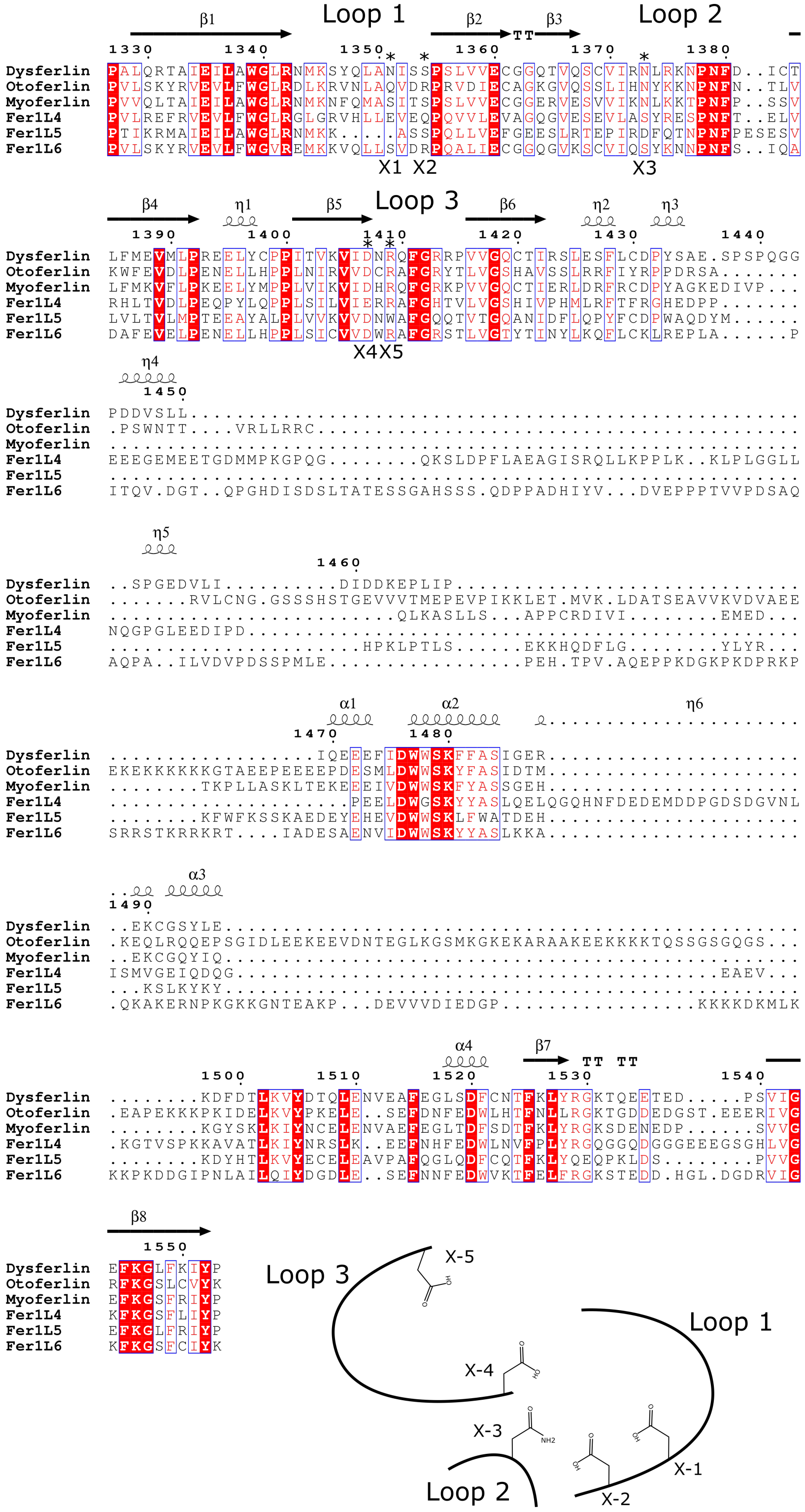

S13 Fig. Model-based primary sequence alignment of the C2E domains of the ferlin family. Secondary structure assignments are based on the known and predicted structures of dysferlin C2E as calculated by DSSP. α-helices are depicted as a

generic helical cartoon. β-strands are rendered as arrows and strict β-turns as TT letters. Residues that are absolutely conserved between all C2D domains are highlighted in red. Conserved residues are boxed in blue. Numbers along the top of the alignment correspond to residue numbers in the human dysferlin sequence. The mean evolutionary relatedness between the C2E domains of all ferlins is 54.1% mean sequence similarity and 34.7% mean identity. A schematic of the calcium ion binding region of the C2E domain is shown below the alignment. X-1, X-2, X-3, X-4, and X-5 demarcate the positions of the known Ca^{2+} binding residues in generic C2 domains. No coordinated calcium ions are shown in C2E as the binding residues are not uniformly present.

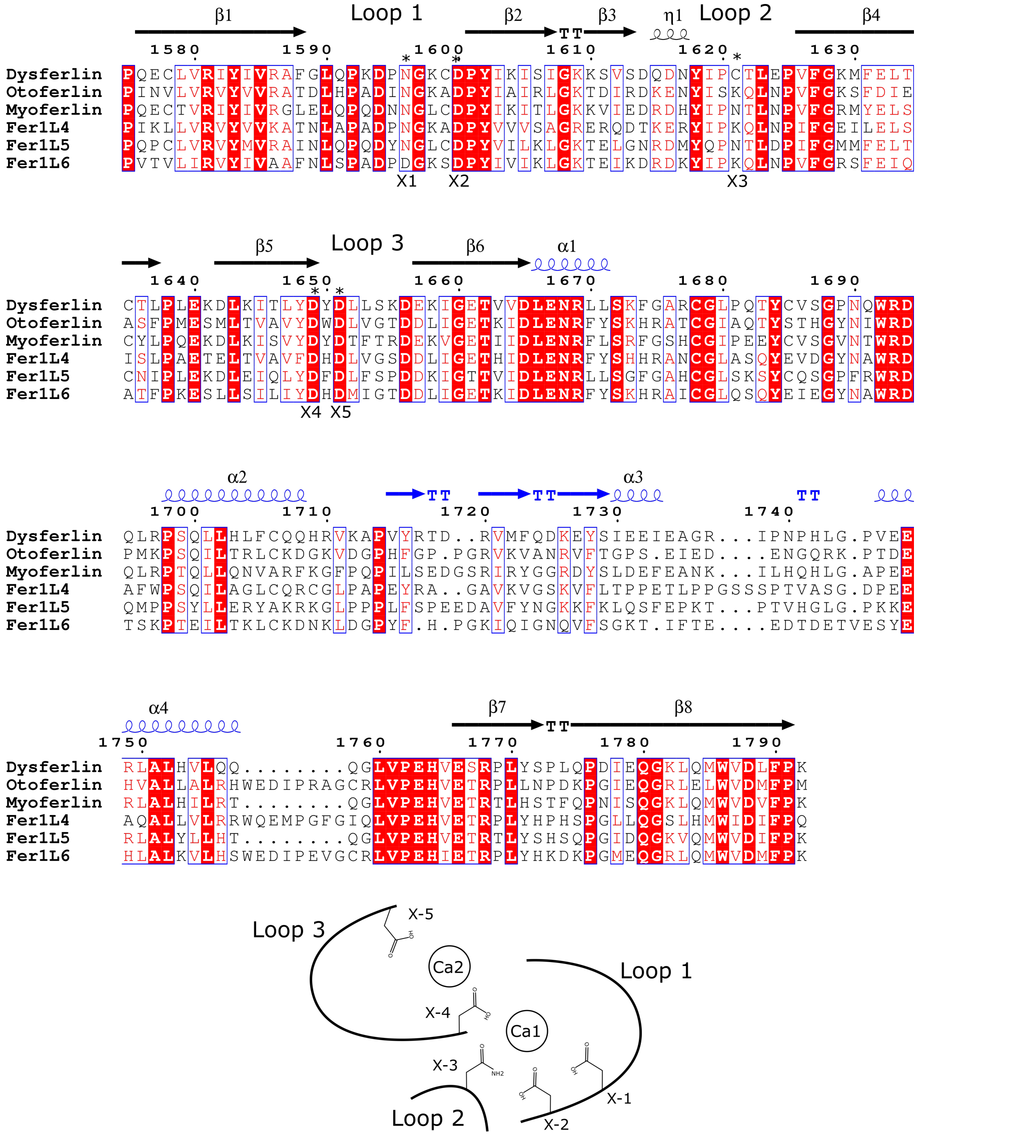

S14 Fig. Model-based primary sequence alignment of the C2F domains of the ferlin family. Secondary structure assignments are based on the predicted structures of dysferlin C2F as calculated by DSSP. α-helices are depicted as a generic helical cartoon. β-strands are rendered as arrows and strict β-turns as TT letters. Residues that are absolutely conserved between all C2F domains are highlighted in red. Conserved residues are boxed in blue. Numbers along the top of the alignment correspond to residue numbers in the human dysferlin sequence. β strands are labeled according to the main body of the C2 domain. The accessory domain between β-6 and β-7 are colored in blue. The mean evolutionary relatedness between the six C2F domains shows 66.7% mean sequence similarity and 48.2% mean identity for all six C2F domains. A schematic of the calcium ion binding region of the C2F domain is shown below the alignment. X-1, X-2, X-3, X-4, and X-5 demarcate the positions of the known Ca^{2+} binding residues in generic C2 domains.

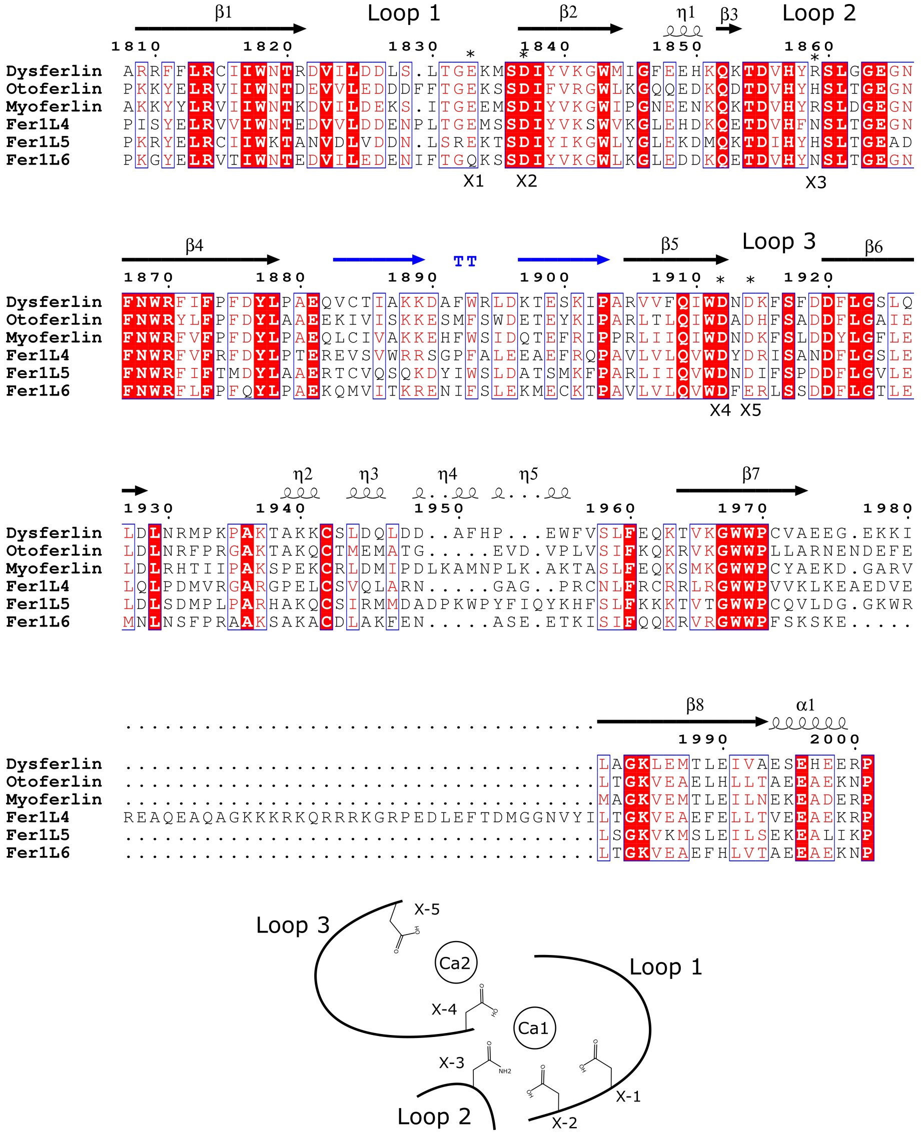

S15 Fig. Model-based primary sequence alignment of the C2G domains of the ferlin family. Secondary structure assignments are based on the known and predicted structures of dysferlin C2G as calculated by DSSP. α-helices are depicted as a generic helical cartoon. β-strands are rendered as arrows and strict β-turns as TT letters. Residues that are absolutely conserved between all C2G domains are highlighted in red. Conserved residues are boxed in blue. Numbers along the top of the alignment correspond to residue numbers in the human dysferlin sequence. The mean evolutionary relatedness between the six C2G domains shows 69.5% mean sequence similarity and 51.2% mean identity for all six C2G domains. A schematic of the calcium ion binding region of the C2G domain is shown below the alignment. X-1, X-2, X-3, X-4, and X-5 demarcate the positions of the known Ca^{2+} binding residues in generic C2 domains.

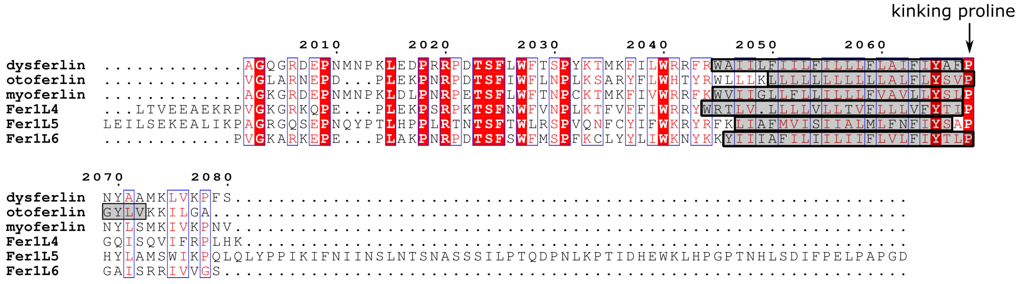

S16 Fig. Residues that extend from the C-terminus of C2G to the C-terminus of the ferlin. The predicted transmembrane residues are highlighted in grey.

```python
import sys
sys.path.append("/home/PyRosetta4") #update with path to pyrosetta
from pyrosetta import *
from pyrosetta.toolbox import *
init()

inputfile = sys.argv[1]
if inputfile == '-h'or inputfile == '':
  print('usage: python3 <input.pdb> <number of relaxes for max>')
else:
  pose = pose_from_pdb(inputfile)
  rounds = int(sys.argv[2])

pose = pose_from_pdb(inputfile)
pose1 = pose_from_pdb(inputfile)

scorefxn = get_fa_scorefxn()
Relax_scores = []
Relax_scores.append(scorefxn(pose))
n=0
while n < rounds:
  relax = pyrosetta.rosetta.protocols.relax.FastRelax()
  relax.set_scorefxn(scorefxn)
  relax.apply(pose)
  Relax_scores.append(scorefxn(pose))
  print(Relax_scores)
  m = n+1
  if round(Relax_scores[m], 4) == round(min(Relax_scores), 4):
    pose_max = pose
    print('the most ' + str(m))
  else:
    pose = pose1
    print('not the most')
  n+=1

print(Relax_scores)

pose_max.dump_pdb('MaxRelaxed_' + inputfile)

output_object = open('Energy_scores.list', 'a')
output_object.write('initial score of ' + inputfile + ': ' + str(
    Relax_scores[0]) + '\nThe Relaxed scores are ' + inputfile + ': ' +
    str(Relax_scores))
output_object.write('\nThe Best Relaxed score is ' + str(min(Relax_scores)
    ) + '\nThe per Residue Score is ' + str(min(Relax_scores)/(pose_max.
    total_residue())) +'\n')
output_object.close()
```

S17 Fig. Energy minimization script used to relax the *in silico* models.

References

1. Li Z, Jaroszewski L, Iyer M, Sedova M, Godzik A. FATCAT 2.0: towards a better understanding of the structural diversity of proteins. Nucleic Acids Research. 2020;48(W1):W60–W64. doi:10.1093/nar/gkaa443.

2. Janson G, Paiardini A. PyMod 3: a complete suite for structural bioinformatics in PyMOL. Bioinformatics. 2020;37(10):1471–1472. doi:10.1093/bioinformatics/btaa849.
