# Supplementary material for: Redefining the architecture of ferlin proteins: Insights into multi-domain protein structure and function
Source: PLoS One. 2022 Jul 28;17(7):e0270188. doi: 10.1371/journal.pone.0270188 (PMC9333456; doi:10.1371/journal.pone.0270188)
Supplement: S1 Raw image — (PDF) [file pone.0270188.s002.pdf]

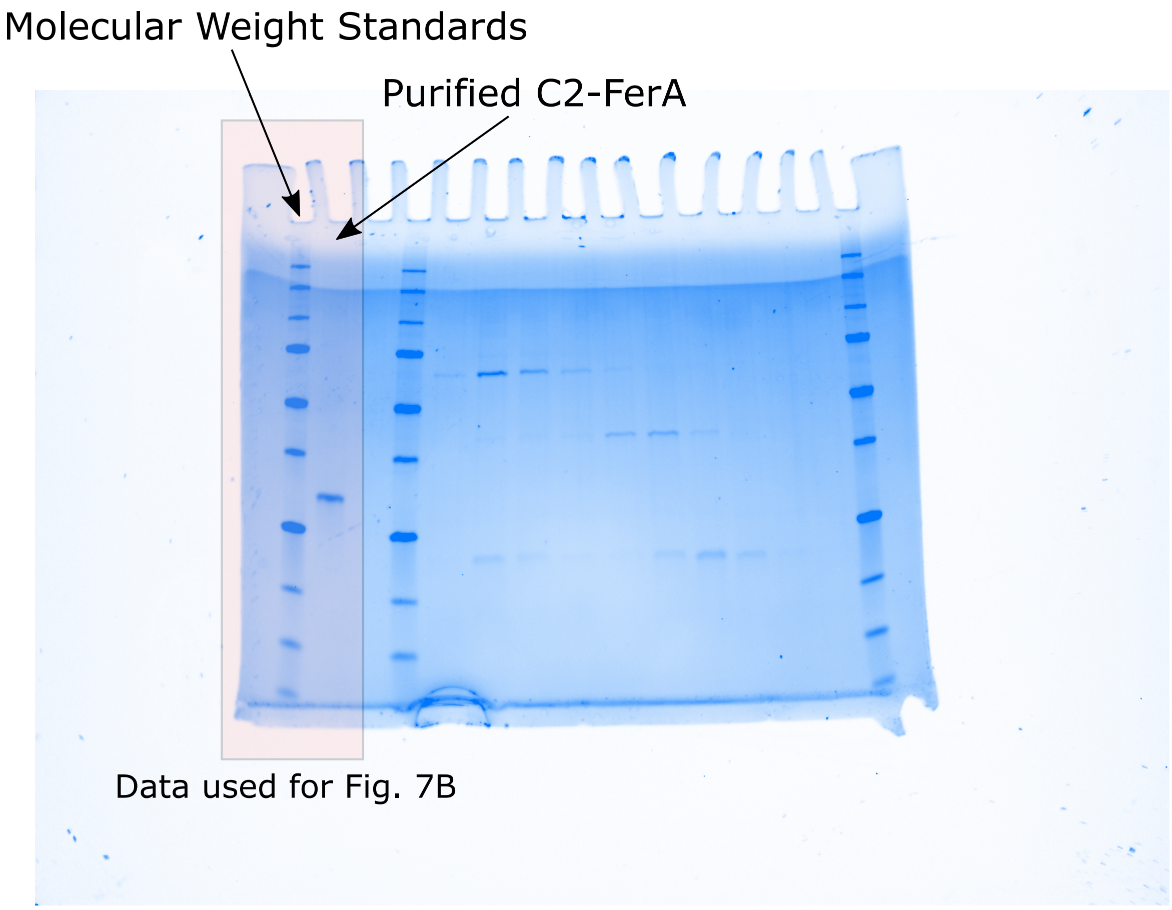

Purity was assessed using SDS PAGE Any-kD Mini-PROTEAN TGX Stain-Free gels from Bio-Rad. The remaining portion of the PAGE gel is from another un-related and unpublished experiment.
